# Host Immune Response Modulation in Avian Coronavirus Infection: Tracheal Transcriptome Profiling In Vitro and In Vivo

**DOI:** 10.3390/v16040605

**Published:** 2024-04-14

**Authors:** Kelsey O’Dowd, Ishara M. Isham, Safieh Vatandour, Martine Boulianne, Charles M. Dozois, Carl A. Gagnon, Neda Barjesteh, Mohamed Faizal Abdul-Careem

**Affiliations:** 1 Health Research Innovation Centre, Faculty of Veterinary Medicine, University of Calgary, Calgary, AB T2N 4N1, Canada; kelsey.odowd@ucalgary.ca (K.O.); fathimaishara.muhamm@ucalgary.ca (I.M.I.); 2 Department of Animal and Poultry Science, Islamic Azad University, Qaemshahr Branch, Qaem Shahr 4765161964, Iran; svatandour@gmail.com; 3 Swine and Poultry Infectious Diseases Research Centre–Fonds de Recherche du Québec (CRIPA-FRQ), Faculty of Veterinary Medicine, Université de Montréal, Saint-Hyacinthe, QC J2S 2M2, Canada; martine.boulianne@umontreal.ca (M.B.); charles.dozois@inrs.ca (C.M.D.); carl.a.gagnon@umontreal.ca (C.A.G.); neda.barjesteh@zoetis.com (N.B.); 4 Department of Clinical Sciences, Faculty of Veterinary Medicine, Université de Montréal, Saint-Hyacinthe, QC J2S 2M2, Canada; 5 Institut National de Recherche Scientifique-Centre Armand-Frappier Santé Biotechnologie, Laval, QC H7V 1B7, Canada; 6 Molecular Diagnostic and Virology Laboratories, Centre de Diagnostic Vétérinaire de l’Université de Montréal (CDVUM), Faculty of Veterinary Medicine, Université de Montréal, Saint-Hyacinthe, QC J2S 2M2, Canada

**Keywords:** transcriptome, tracheal epithelial cell, trachea, infectious bronchitis virus, chicken, immune response

## Abstract

Infectious bronchitis virus (IBV) is a highly contagious *Gammacoronavirus* causing moderate to severe respiratory infection in chickens. Understanding the initial antiviral response in the respiratory mucosa is crucial for controlling viral spread. We aimed to characterize the impact of IBV Delmarva (DMV)/1639 and IBV Massachusetts (Mass) 41 at the primary site of infection, namely, in chicken tracheal epithelial cells (cTECs) in vitro and the trachea in vivo. We hypothesized that some elements of the induced antiviral responses are distinct in both infection models. We inoculated cTECs and infected young specific pathogen-free (SPF) chickens with IBV DMV/1639 or IBV Mass41, along with mock-inoculated controls, and studied the transcriptome using RNA-sequencing (RNA-seq) at 3 and 18 h post-infection (hpi) for cTECs and at 4 and 11 days post-infection (dpi) in the trachea. We showed that IBV DMV/1639 and IBV Mass41 replicate in cTECs in vitro and the trachea in vivo, inducing host mRNA expression profiles that are strain- and time-dependent. We demonstrated the different gene expression patterns between in vitro and in vivo tracheal IBV infection. Ultimately, characterizing host–pathogen interactions with various IBV strains reveals potential mechanisms for inducing and modulating the immune response during IBV infection in the chicken trachea.

## 1. Introduction

Infectious bronchitis virus (IBV) is a highly contagious virus that causes mild to severe respiratory infections in chickens. The severity of the disease is dependent on several factors, such as environment, IBV strain, vaccination program, and coinfections [1]. The resulting disease is known as infectious bronchitis (IB) and is characterized by tracheitis and loss of ciliary activity in the upper respiratory tract of chickens [2]. Chickens of all ages are susceptible to IBV infection; however, the disease is more severe in young chicks [3]. IB is an acute disease transmitted via the respiratory tract by inhalation or by direct contact with contaminated poultry, litter, or equipment. The incubation period is short, 18 to 36 h, and clinical signs develop around 24 to 48 h post exposure [3,4]. Clinical manifestations of the respiratory tract include sneezing, gasping, coughing, tracheal rales, nasal discharge, and dyspnea [5]. In older chickens and in laying hens, respiratory signs can be mild or even absent [1]. Although initial infection typically occurs in the epithelial layer of the upper respiratory tract, IBV can disseminate and infect the gastrointestinal, renal, reproductive, and immune systems [5,6,7,8,9,10], potentially via the lymph or blood [6,11,12]. Depending on the IBV strain, this can lead to other clinical and pathological manifestations, such as nephritis [13], a decline in egg production and quality of the egg and egg shell in layer/breeder flocks [14,15,16], and a depletion of immune cells [7].

IBV is a positive-sense, single-stranded RNA virus and, typical of many RNA viruses, it is associated with rapid mutation rates and recombination in the genome, leading to the emergence of genetically diverse strains at a global level [17,18]. Vaccination with live attenuated/killed vaccines is one of the most important methods for the control of IB, along with rigorous biosecurity measures, but the aforementioned genetic diversity of these viruses is a significant obstacle for efficient and effective protection of flocks from potential outbreaks, as there is poor cross protection between heterologous strains [19]. A novel IBV variant, IBV Delmarva (DMV)/1639, emerged in 2011 [20]. Since 2015, IBV DMV/1639 strains have become more prevalent in Eastern Canada, namely in Quebec and Ontario [21,22,23]. Recent work has been conducted to characterize the underlying immunopathogenesis of this Canadian IBV DMV/1639 strain [21,24,25]. This DMV strain, among other IBV strains, such as the Massachusetts (Mass)-type IBVs, have been associated with the failure of a previously infected flock to reach peak lay due to a variable number of birds with severe developmental oviduct lesions, also known as false layers [14,23,24,26,27,28]. In addition, flock depopulation and secondary bacterial infections of the respiratory system following IBV infection cause significant economic losses to the poultry industry [1]. This highlights the importance in understanding the detailed mechanism of pathogenesis and host defense during IBV infection at the primary site of infection, namely, the airway epithelial cells.

The chicken immune system is a complex system designed to fight off invading pathogens, including viruses such as IBV. When the virus crosses the primary mucosal barriers, the innate immune responses provide the first line of defense and the airway epithelial cells become the primary target for the pathogen. In birds and mammals alike, airway epithelial cells have many important immune functions, which include the secretion of antimicrobial substances, cytokines and growth factors, cell-to-cell communication with immune cells, and modulation of early adaptive immunity during viral infections [29,30]. The induction of the innate response is dependent on many factors, including the detection of viral pathogen-associated molecular patterns (PAMPs) through pattern recognition receptors (PRRs), including Toll-like receptors (TLRs) [31]. The primary antiviral innate immune responses are characterized by this recognition and activation, resulting in the transcriptional activation of type I interferons (IFNs) and IFN-stimulated genes (ISGs), such as IFN-induced proteins with tetratricopeptide repeats (IFIT), myxovirus-resistance protein (MX), protein kinase R (PKR), and 2′-5′ oligoadenylate synthase-like (OASL) proteins [32,33]. These proteins are important for protecting the host and conferring resistance to RNA viral infections [34,35]. On the other hand, IBV has been shown to inhibit type 1 IFN response in primary chicken renal and tracheal epithelial cells and a chicken fibroblast cell line [36].

Since the early 2000s, researchers have aimed to map the host gene expression patterns involved in IBV infection [37,38,39]. More recent transcriptomic studies have looked at chicken spleen tissues [40,41,42], tracheal tissues [43,44,45], lung tissues [41], human lung epithelial-like cells [46], chicken kidney tissues and cells [47,48,49,50,51], dendritic cells [52,53], macrophages [54], and fibroblasts [55] upon infection with various strains of IBV. Currently, there are no RNA-seq studies specifically looking at IBV infection in chicken tracheal epithelial cells (cTECs), nor using the IBV DMV/1639 strain, which has been the dominant IBV genotype circulating in Canada [21,22] and the United States of America (USA) in recent years [20,56]. Despite extensive research on the pathogenicity of these different strains of the virus [7,24,28,57,58,59,60], there is a lack of knowledge regarding the regulation of molecular mechanisms involved in the initial induction of the host antiviral responses at the level of the trachea and tracheal epithelial cells upon infection with different strains of IBV, which may help to explain the differing pathogenesis in the tracheal tissues of infected birds. To this end, we aimed to characterize the impact of IBV DMV/1639 and IBV Mass41 at the primary site of infection, namely, in cTECs in vitro and the trachea in vivo, and to evaluate the impact of infection on the host gene expression. We hypothesized that the host antiviral reactions elicited by IBV DMV/1639 and IBV Mass41 exhibit unique characteristics in terms of differential expression of immune-related genes in the infection models presented in this study.

## 2. Materials and Methods

### 2.1. Virus Propagation and Titration

The Canadian IBV DMV/1639 clinical isolate IBV/Ck/Can/17-036989 (GenBank accession no. MN512435), isolated from the kidneys of infected layers (Ontario, Canada) [21], and the Canadian IBV Mass41 clinical isolate IBV/Ck/Can/21-2455844 (GenBank accession no. PP373115), obtained from a pool of tissues from infected broilers (Quebec, Canada) (Dr. Carl A. Gagnon, CDVUM), were propagated by inoculation in 10-day-old specific-pathogen-free (SPF) embryonated chicken (layer chickens, white Leghorn) eggs obtained from the Canadian Food Inspection Agency (CFIA), Ottawa, ON, Canada [61,62]. Allantoic fluid was harvested at 3 days post-infection (dpi) and viral titers were determined by 50% embryo infectious dose (EID_50_), as described previously [21,61]. The viral titer was calculated using the Reed and Muench method and expressed as EID_50_/mL [63]. The viral titers were determined to be 10^6.0^ EID_50_/mL for IBV DMV/1639 and 10^6.5^ EID_50_/mL for IBV Mass41.

### 2.2. cTEC Preparation

Primary cTEC isolation was performed as previously described with some modifications [64,65,66]. Briefly, tracheas were aseptically dissected from 19-day-old SPF chicken embryos (CFIA, Ottawa, ON, Canada) and digested with filter-sterilized protease from Streptomyces griseus (Pronase, Sigma-Aldrich Oakville, ON, Canada) (2 mg/mL) in complete Medium 199 (Sigma-Aldrich Oakville, ON, Canada) supplemented with 2 mM GlutaMax supplement, 25 mM 4-(2-hydroxyethyl)-1-piperazineethanesulfonic acid (HEPES) buffer, 100 U/mL penicillin/100 µg/mL streptomycin, 50 µg/mL gentamicin, and 0.25 µg/mL amphotericin B (Gibco, Burlington, ON, Canada). The cells were treated with a filter-sterilized 0.5 mg/mL DNase solution (Deoxyribonuclease I from bovine pancreas, Sigma-Aldrich, Oakville, ON, Canada) in complete Medium 199, followed by a brief incubation period in complete Dulbecco’s Modified Eagle Medium/Nutrient Mixture F-12 (DMEM/F-12), containing 10% FBS, 2 mM GlutaMax supplement, 100 U/mL penicillin/100 µg/mL streptomycin, 50 µg/mL gentamicin, 0.25 µg/mL amphotericin B, 1 mM β-mercaptoethanol (BME), and 1% non-essential amino acids (MEM NEAA) (Gibco, Burlington, ON, Canada), as a negative selection step for fibroblast growth. Finally, the cells were resuspended in complete DMEM/F-12 medium supplemented with 10% chicken embryo extract. The chicken embryo extract was prepared in-house from 11-day-old SPF chicken embryos as previously described in the protocol developed by Pajtler and colleagues [67]. The cTECs were seeded at a viable cell density (determined by trypan blue exclusion test) of 3 × 10^5^ cells per well into wells of 5% MatriGel-coated (Corning Inc., Corning, NY, USA) 24-well culture plates. After 4 days of incubation at 37 °C 5% CO_2_, the cells were subjected to further experiments as described in Section 2.3 below.

### 2.3. Infection of cTECs with IBV

Tracheal epithelial cells were cultured in complete DMEM/F-12 (serum-free) infection medium containing 2 mM GlutaMax supplement, 100 U/mL penicillin/100 µg/mL streptomycin, 50 µg/mL gentamicin, 25 mM HEPES buffer, and 2.5% bovine serum albumin (BSA 7.5% solution) (Gibco, Burlington, ON, Canada) and incubated at 37 °C 5% CO_2_ for all steps. Prior to infection, cells were washed twice with medium and then infected with 200 µL with a low (2 × 10^4^ EID_50_/mL), intermediate (1 × 10^5^ EID_50_/mL), or high (5 × 10^5^ EID_50_/mL) dose, diluted in phosphate-buffered saline (PBS), of either IBV DMV/1639 or IBV Mass41. The control groups received DMEM/F-12 infection medium only. Subsequently, cells were washed twice two hours post-infection (hpi) following the adsorption period and incubated in fresh DMEM/F-12 infection medium. These doses were selected in part based on a previous study [65]. At 0, 18, 24, and 48 h, supernatants were collected in TRIzol™ LS reagent (Invitrogen, Burlington, ON, Canada), to determine viral genome load. Based on the results of this preliminary study, in a separate experiment, the cells were infected with the different IBV isolates at a high (5 × 10^5^ EID_50_/mL) dose and the cells were collected in QIAzol™ reagent (QIAGEN, Toronto, ON, Canada) at an early time point, 3 h, and at a later time point near the peak of viral genome load detected, 18 h, for RNA sequencing.

### 2.4. Chickens

One-day-old SPF chickens (layer chickens, white Leghorn) (*n* = 60) were purchased from the CFIA, Ottawa, ON, and housed and closely monitored in the animal facilities by staff at the National Experimental Biology Laboratory (NEBL) of the Institut national de la recherche scientifique (INRS) Armand-Frappier Santé Biotechnologie Research Centre, where the experiments were conducted in temperature-controlled poultry isolators in negative pressure rooms. The chickens were divided into 5 groups (*n* = 12 chickens/group). The groups were named as follows: IBV DMV/1639 low dose, IBV DMV/1639 high dose, IBV Mass41 low dose, IBV Mass41 high dose, and uninfected control. The experimental protocols were approved by the Institutional Animal Care and Use Committee (IACUC) of the Université de Montréal (ethics protocol no. 21-Rech-2120) and the INRS (ethics protocol no. 2106-03). The tracheal tissue samples used for the real-time quantitative polymerase chain reaction (qPCR) mRNA gene expression validation experiments were from chickens that were housed at the Veterinary Science Research Station (VSRS), Spyhill, Campus, University of Calgary, and subjected to the same experimental conditions as those in the NEBL INRS Armand-Frappier Santé Biotechnologie Research Centre animal facility. The experimental protocols for these experiments were approved by the Veterinary Science Animal Care Committee (VSACC) and the Health Science Animal Care Committee (HSACC) of the University of Calgary (ethics protocol no. AC22-0012).

### 2.5. Infections of Chickens with IBV

The IBV stocks were diluted in PBS to the appropriate doses for inoculation. Six-day-old SPF chickens were inoculated with a low dose (10^4^ EID_50_/bird) or a high dose (10^5^ EID_50_/bird) of either IBV DMV/1639 or IBV Mass41 through the intranasal and intraocular routes (100 µL). The negative control group received PBS. Samples from the upper half of the trachea were collected at 4 (*n* = 6 chickens/group) and 11 dpi (*n* = 6 chickens/group) and stored in RNAlater^®^ (Invitrogen, Burlington, ON, Canada).

### 2.6. Quantification of IBV Viral Genome Load and Host mRNA Gene Expression

From cTEC cell culture supernatants, total RNA was extracted from the samples using the TRIzol™ LS reagent (Invitrogen, Burlington, ON, Canada), according to the manufacturer’s protocol. For the tracheas collected from IBV DMV/1639-infected chickens at 4 dpi and 11 dpi, the samples were lysed in TRIzol™ reagent (Invitrogen, Burlington, ON, Canada) and homogenized using 0.5 mm glass beads and a tissue homogenizer (MP FastPrep-24 Classic Instrument, MP Biomedicals, Solon, OH, USA). Total RNA was extracted according to the manufacturer’s protocol. Isolated RNA was resuspended in 20 µL RNase-free water. Assessment of RNA concentration and quality was performed using the NanoDrop ND-1000 spectrophotometer (Thermo Scientific, Wilmington, DE, USA). Using the High-Capacity Reverse Transcription Kit with random primers (Applied Biosystems, Waltham, MA, USA) according to manufacturer’s instructions, complementary deoxyribonuclease (cDNA) synthesis was performed for 500 ng (cTEC supernatants) or 2000 ng (tracheas) of RNA per sample. qPCR targeting the IBV nucleoprotein gene (N) was performed for quantification of IBV viral genome load in cTEC supernatants and trachea and for host mRNA gene expression in the tracheal tissues, using gene-specific primers (Table S1, [68,69,70,71,72,73,74,75]) at a final concentration of 5 nM (Sigma-Aldrich, Saint-Louis, MO, USA) and PowerUp SYBR Green Master Mix (Applied Biosystems, Burlington, ON, Canada) in a 20 µL reaction according to the manufacturer’s instructions. Furthermore, a 10-fold dilution series of the IBV-N gene plasmid was used to generate the standard curve, as previously described [68]. The IBV-N gene plasmid DNA was generated from a stock prepared in-house. IBV-N plasmid transformation was performed using the Subcloning Efficiency™ DHα Competent cells (Thermo Scientific, Burlington, ON, Canada) and purification was performed using the GeneJET Plasmid miniprep kit (Thermo Scientific, Burlington, ON, Canada), according to manufacturers’ instructions. The qPCR cycling program for quantification of all genes consisted of a pre-incubation at 95 °C for 20 s, and amplification/extension at 95 °C for 3 s and 60 °C for 30 s, repeated for 40 cycles. Melting curve analysis was assessed at 95 °C for 10 s (segment 1), 65 °C for 5 s (segment 2), and 9 °C for 5 s (segment 3). Fluorescence acquisition was performed at 60 °C for 30 s and the results for IBV genome load are presented as log_10_ IBV genome copies per 1 μL of reaction/cDNA [68]. Fold-changes for host mRNA gene expression were calculated using the 2^−ΔΔCt^ method [76] and quantified relative to the β-actin housekeeping gene.

### 2.7. RNA Isolation, cDNA Library Preparations and High-Throughput Sequencing

For the RNA-sequencing (RNA-seq) experiments, total RNA was isolated from cTECs, and lysed and homogenized tracheal tissues using QIAzol™ reagent (QIAGEN, Toronto, ON, Canada) and the miRNeasy Mini Kit (QIAGEN, Toronto, ON, Canada) according to the miRNeasy Mini Kit Quick-Start protocol. The purified RNA was eluted in 30 µL RNase-free water. Prior to sequencing, RNA quality control was performed by automatic electrophoresis-based analysis (TapeStation RNA Screen Tape, Agilent, Santa Clara, CA, USA).

For the cTEC samples, RNA library preparations and sequencing were performed at Plateforme de séquençage de nouvelle génération of the Université Research Center of the CHU de Québec-Université Laval. Twenty-four libraries were prepared for RNA-seq, with 4 replicates per treatment group: IBV DMV/1639 3 h, IBV DMV/1639 18 h, IBV Mass41 3 h, IBV Mass 18 h, control (CTRL) 3 h, CTRL 18 h. Each replicate consisted of a pool of cells from 2 individual embryos. Infected samples are from cTECs infected with a high dose (5 × 10^5^ EID_50_/mL) of IBV.

For the tracheal samples, RNA library preparations and sequencing were performed at the McGill Applied Genomics Innovation Core (MAGIC) of the McGill Genome Centre, McGill University. Eighteen libraries were prepared for RNA-seq, with 3 replicates per treatment group: IBV DMV/1639 4 dpi, IBV DMV/1639 11 dpi, IBV Mass41 4 dpi, IBV Mass41 11 dpi, control (CTRL) 4 dpi, CTRL 11 dpi. Each replicate consisted of a pool of tracheal tissue from 2 individual chickens. Infected samples were from tracheal tissues that originated from chickens infected with a high dose (10^5^ EID_50_/bird) of IBV. The RNA libraries were sequenced on a NovaSeq 6000 S4 (Illumina, San Diego, CA, USA) platform to generate 100 base pair (bp) paired-end reads.

### 2.8. RNA-Seq Differential Expression, Gene Ontology (GO), and Pathway Analysis

Analysis for RNA-seq data was performed using the open-source framework GenPipes [77]. Analyses were conducted using RStudio [78,79], unless stated otherwise. The R packages knitr [80], ggrepel [81], tibble [82], tidyverse [83], magrittr [84], hablar [85], and kableExtra [86] were used for analysis and formatting. RNA-SeQC [87] was used to assess the quality of the generated reads. Trimmomatic [88] was used to process raw sequencing reads and trim adaptor sequences and low-quality score-containing bases (Phred score < 30) from reads. The resulting reads were aligned to the Ensembl chicken (Gallus gallus) reference genome (ASM223467v1, GRCg6a, INSDC Assembly GCA_000002315.5) from http://aug2020.archive.ensembl.org/Gallus_gallus/Info/Index (accessed on 7 June 2022). This was conducted using Spliced Transcripts Alignment to a Reference (STAR) [89] and read counts were obtained using HTSeq [90]. The R package DESeq2 [91] was then used to identify differences in expression levels between the groups using negative binomial generalized linear model (GLM) fitting and Wald statistics: nbinomWaldTest. Data were batched normalized and log transformed. The R package “ashr” [92] was used to shrink log_2_ fold-changes (log_2_FC) for gene expression data. For the purpose of this study, differential gene expression was based on an infected group compared to the uninfected control group at the same time point and genes were considered differentially expressed (DE) if the adjusted *p*-value was <0.05 and log_2_FC was ≥|1| or fold-change (FC) ≥ |2|. Principle Component Analysis (PCA) plots, heatmaps using the R packages ComplexHeatmap [93] and tidyHeatmap [94], and volcano plots using the R package EnhancedVolcano [95] were created in R [78,79]. Venn diagram analysis and visualization were created using the online tools https://bioinformatics.psb.ugent.be/webtools/Venn/ and Venny (accessed on 9 January 2024) [96].

All genes that were DE were considered (separated by down- and up-regulated genes) for further analyses. GO functional enrichment analyses, or over-representation analyses (ORA), and visualizations for Biological Process (BP), Molecular Function (MF), and Cellular Component (CC) were performed using the R packages gprofiler2 (g:Profiler) [97,98], enrichplot [99], DOSE [100], and ggplot2 [101]. Enrichment *p*-values were based on a hypergeometric test, the g:GOSt method, using the default g:SCS method applied for multiple testing correction. This corresponds to an experiment-wide threshold of α = 0.05, wherein at least 95% of matches above the threshold are statistically significant. The background used was the set of known genes and terms with GO evidence codes Inferred from Electronic Annotation (IEA) were excluded. The R package GOfuncR [102] was used to investigate relationships between enriched GO term parent and child nodes. Kyoto Encyclopedia of Genes and Genomes (KEGG) [103] pathway analysis and visualization for key enriched pathways was performed using the R packages gprofiler2 (g:Profiler) [97,98], pathview [104], and org.Gg.eg.db [105].

### 2.9. Statistical Analysis

Statistical analysis for IBV genome loads for each strain was assessed using two-way analysis of variance (ANOVA), followed by Tukey’s post hoc test. The differences were considered significant if the *p*-value was <0.05. Statistical analysis was performed using GraphPad Prism 10 software (GraphPad, La Jolla, CA, USA). Statistical methods for sequence data analysis are contained within the software used.

## 3. Results

### 3.1. IBV Genome Load in cTEC Supernatants and the Trachea

**Figure 1:**
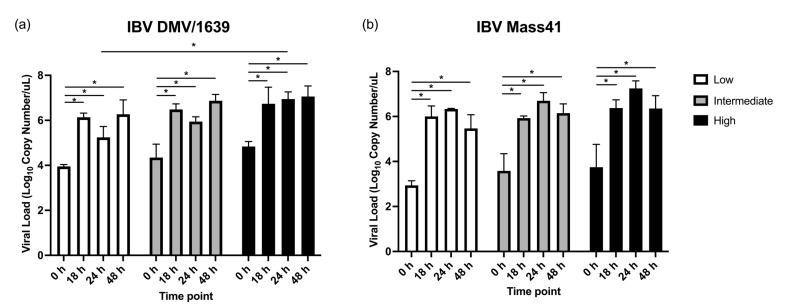
IBV genome load in the supernatants of cTECs infected with IBV DMV/1639 or IBV Mass41 strains. The cTECs were inoculated with a low (2 × 10^4^ EID_50_/mL), intermediate (1 × 10^5^ EID_50_/mL), or high (5 × 10^5^ EID_50_/mL) dose of either IBV DMV/1639 (**a**) or IBV Mass41 (**b**). At 0, 18, 24, and 48 h, supernatants were collected, RNA extracted, and cDNA synthesized to determine viral genome loads using a qPCR assay. Statistical analysis for IBV viral genome loads for each strain was assessed using two-way ANOVA followed by Tukey’s post hoc test. Significant differences (*p*-value < 0.05) are denoted by *. The error bars represent standard deviation (SD).

The effects of the different doses and time points on IBV genome loads in the cTEC supernatants were assessed by qPCR for both IBV strains and are shown in Figure 1. No IBV genome was detected for the uninfected controls. Upon cTEC infection with different doses of IBV DMV/1639 (Figure 1a) or IBV Mass41 (Figure 1b), it was found that there was a significant increase in IBV genome load between the time point 0 h and the time points 18 h, 24 h, and 48 h for the three doses evaluated (*p*-value < 0.05). No significant differences were observed between the time points 18 h, 24 h, and 48 h within each respective dose (*p*-value > 0.05). In addition, a significantly higher IBV genome load was observed with the IBV DMV/1639 high dose group compared to the low dose group at 24 h (*p*-value < 0.05).

**Figure 2:**
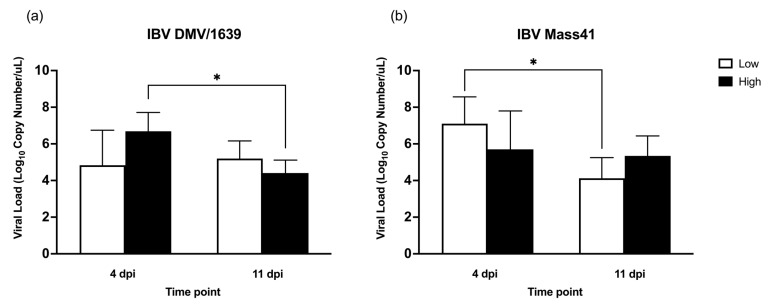
IBV viral genome in tracheal tissues from chickens infected with IBV DMV/1639 or IBV Mass41. Six-day-old chickens were infected with a low (10^4^ EID_50_/bird) or a high (10^5^ EID_50_/bird) dose of either IBV DMV/1639 (**a**) or IBV Mass41 (**b**). At 4 dpi and 11 dpi, tracheal tissue samples were collected, RNA extracted, and cDNA synthesized to determine viral genome load using a qPCR assay. Statistical analysis for differences in IBV genome loads for each strain was conducted using two-way ANOVA followed by Tukey’s post hoc test, and significant differences (*p*-value < 0.05) are denoted by *. The error bars represent SD.

The IBV genome loads in the trachea samples collected during the in vivo experiment are shown in Figure 2. The samples from all infected groups were IBV-positive. No IBV was detected in uninfected controls at 4 dpi and 11 dpi. The IBV genome load in the trachea was significantly higher (*p*-value < 0.05) in high dose IBV DMV/1639-infected chickens at 4 dpi compared to 11 dpi (Figure 2a). In the IBV Mass41-infected group, there was a significant decrease (*p*-value < 0.05) in viral genome load from 4 dpi to 11 dpi in the tracheas of chickens challenged with a low dose of virus (Figure 2b).

### 3.2. mRNA Expression and Functional Profiles from cTECs Infected with Different IBV Strains

The mRNA expression profiles of cTECs infected with the high dose (5 × 10^5^ EID_50_/mL) of IBV DMV/1639 or IBV Mass41 at 3 h and 18 h were evaluated to determine strain-specific and temporal-related changes in gene expression. All RNA-seq differential expression results are compiled in Table S2, which includes the following comparisons: CTRL 18 h vs. CTRL 3 h, IBV DMV/1639 3 h vs. CTRL 3 h, IBV DMV/1639 18 h vs. CTRL 18 h, IBV DMV/1639 18 h vs. IBV DMV/1639 3 h, IBV Mass41 3 h vs. CTRL 3 h, IBV Mass41 18 h vs. CTRL 18 h, IBV Mass41 18 h vs. IBV Mass41 3 h. For this study, comparisons of the treatment groups and the control groups at the same respective time point were considered (IBV DMV/1639 3 h vs. CTRL 3 h, IBV DMV/1639 18 h vs. CTRL 18 h, IBV Mass41 3 h vs. CTRL 3 h, IBV Mass41 18 h vs. CTRL 18 h). The results filtered for significantly DE mRNAs (defined by an adjusted *p*-value < 0.05 and a log_2_FC ≥ |1|) are summarized in Table S3.

**Figure 3:**
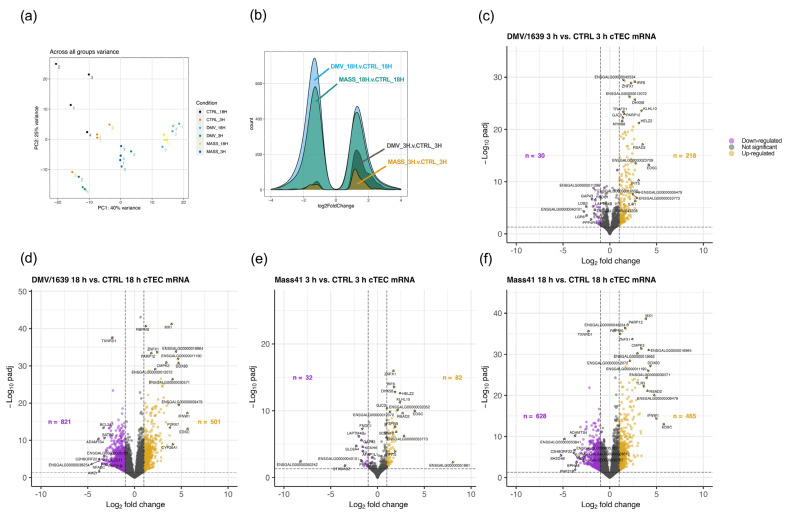
Differential expression of mRNAs from cTECs infected with IBV DMV/1639 or IBV Mass41. The PCA plot (**a**) evaluates the variance across all samples based on the log counts of all mRNAs. The histogram (**b**) represents the log_2_FC distribution of fluorescence signal intensity ratios for DE mRNAs of cTECs infected with IBV DMV/1639 or IBV Mass41 at 3 h and 18 h. The volcano plots show DE mRNAs of cTECs infected with IBV DMV/1639 at 3 h (**c**) and 18 h (**d**) or IBV Mass41 at 3 h (**e**) and 18 h (**f**), relative to their respective control groups. The horizontal dotted line represents the adjusted *p*-value < 0.05 threshold. The vertical dotted lines represent the log_2_FC ≥ |1| (FC ≥ |2|) threshold. The x-axis limits are set from −10 to 10 log_2_FC. Down-regulated mRNAs are represented by purple data points and up-regulated mRNAs are represented by yellow data points. The list of all up- and down-regulated mRNAs for each treatment group are shown in Table S3.

The variance in log counts across all samples by group is shown in Figure 3a. In addition, the heatmaps provided in the Supplementary Files (Figure S1) demonstrate the relationships between cTECs infected with IBV DMV/1639 at 3 h (Figure S1a) and 18 h (Figure S1b) or IBV Mass41 at 3 h (Figure S1c) and 18 h (Figure S1d), relative to their respective control groups. The clustering is based on the similarity of normalized log counts, rather than differential expression, and there are differences in counts between the virus-treated groups and uninfected control groups. Overall, there are a higher number of DE mRNAs at 18 h as compared to the 3 h groups for both virus strains (Figure 3b). Among all treatment groups, including IBV DMV/1639- and IBV Mass41-infected cTECs at 3 h and 18 h, a total of 1653 DE mRNAs were identified among all treatment groups (Table S3). Figure 3c–f shows the number of down- and up-regulated mRNAs per group which passed the adjusted *p*-value < 0.05 and log_2_FC ≥ |1| thresholds. Briefly, a total of 248 and 1322 DE mRNAs, 30 and 821 down-regulated mRNAs, and 218 and 501 up-regulated mRNAs were identified for IBV DMV/1639 3 h and IBV DMV/1639 18 h, respectively. Furthermore, 114 and 1093 DE mRNAs, 32 and 628 down-regulated mRNAs, and 82 and 465 up-regulated mRNAs were identified for IBV Mass41 3 h and IBV Mass41 18 h, respectively. At the 3 h time point, fewer genes were down-regulated than up-regulated, while at the 18 h time point, more genes were down-regulated than up-regulated.

**Figure 4:**
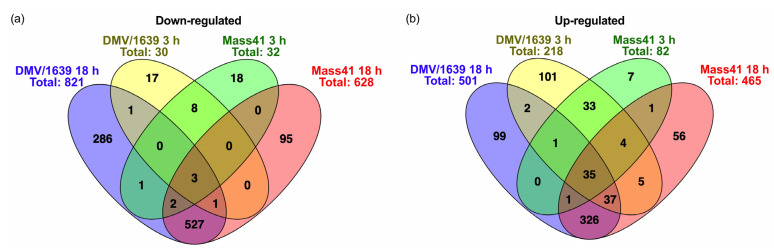
Common and unique DE mRNAs of cTECs infected with IBV DMV/1639 or IBV Mass41. The Venn diagram illustrates common and unique down-regulated (**a**) and up-regulated (**b**) DE mRNAs among cTECs infected with IBV DMV/1639 or IBV Mass41 at 3 h and 18 h. Lists of common and unique DE mRNAs are found in Table S4.

Some DE mRNAs were present in several treatment groups, as shown in Figure 4a, for down-regulated mRNAs, and in Figure 4b for up-regulated mRNAs. Details of the Venn diagram results are summarized in Table S4. There were 3 down-regulated mRNAs, namely solute carrier family 6 member 4 (SLC6A4), Kruppel-like factor (KLF) 1 (KLF1), and ENSGALG00000008599, and 35 up-regulated mRNAs common to all treatment groups (for both IBV strains at both time points). The commonly up-regulated mRNAs among all groups included immune response-related genes zinc finger NFX1-type-containing 1 (ZNFX1), poly(adenosine diphosphate-ribose) polymerase family member 9 (PARP9), deltex E3 ubiquitin ligase 3L (DTX3L), tripartite motif-containing 25 (TRIM25), IFIT5, MX1, OASL, IFN regulatory factor (IRF)7, TLR3, DExH-box helicase 58 (DHX58), also known as Laboratory of Genetics and Physiology 2 (LPG2), IFN induced with helicase C domain 1 (IFIH1), also known as melanoma differentiation-associated protein 5 (MDA5), radical S-adenosyl methionine domain-containing 2 (RSAD2), also known as viperin, and eukaryotic translation initiation factor 2 α kinase 2 (EIF2AK2), also known as PKR. Furthermore, IFN-induced transmembrane protein 3-like (IFITM3) is down-regulated in the IBV DMV/1639 and IBV Mass41 3 h groups but up-regulated in the IBV DMV/1639 and IBV Mass41 18 h groups. In addition, signal transducer and activator of transcription (STAT) 1 (STAT1), STAT2, tumor necrosis factor (TNF) receptor-associated factor (TRAF)-type zinc finger domain-containing 1 (TRAFD1), IFITM5, adenosine deaminase that acts on RNA (ADAR), Moloney leukemia virus 10 (MOV10), and DExD/H box helicase 60 (DDX60) were up-regulated in the IBV DMV/1639 3 h, IBV DMV/1639 18 h, and IBV Mass41 18 h groups, while suppressor of cytokine signaling (SOCS) 1 (SOCS1) was up-regulated in the IBV DMV/1639 3 h, IBV Mass41 3 h, and IBV Mass41 18 h groups. Moreover, myeloid differentiation primary response (MYD)88 was up-regulated in the IBV DMV/1639 3 h and IBV Mass41 18 h groups.

Few common DE mRNAs were identified between time points for the same IBV strains. Protein phosphatase 4 regulatory subunit 4 (PPP4R4) was down-regulated, and two mRNAs, complement component 1r (C1R) and ENSGALG00000046098, were up-regulated in the IBV DMV/1639 3 h and 18 h groups. Furthermore, TNF superfamily member (TNFSF) 15 (TNFSF15) was down-regulated in the IBV DMV/1639 18 h group but up-regulated in the IBV DMV/1639 3 h group. Potassium voltage-gated channel subfamily D member 2 (KCND2) was up-regulated in the IBV Mass41 3 h and 18 h groups. The IBV DMV/1639 3 h and IBV Mass41 3 h groups shared 8 down-regulated mRNAs and 33 up-regulated mRNAs, including IRF1 and IRF8. Of all the intersecting groups, the IBV DMV/1639 18 h and IBV Mass41 18 h groups had the highest number of common DE mRNAs, with 527 down-regulated mRNAs and 326 up-regulated mRNAs.

At 18 h, the common down-regulated mRNAs included interleukins (IL)-1β (IL1B), IL-21 receptor (IL21R), IL-8-like 1 (IL8L1), IL-2 receptor subunit α (IL2RA), IL-31 receptor subunit α (IL3RA), IL-10 receptor subunit α (IL10RA), ISG20, TNF receptor superfamily (TNFRSF) 18 (TNFRSF18), TNFRSF1B, TNFRSF8, TRAF3, tripartite motif-containing 9 (TRIM9), SOCS3, activator protein (AP)-1 transcription factor subunits Jun proto-oncogene (JUN) and Fos proto-oncogene (FOS), and nuclear factor of κ light polypeptide gene enhancer in B-cells (NFKB) inhibitor, α (NFKBIA), also known as IκBα. The common up-regulated mRNAs at 18 h included IFN ω 1 (IFNW1), IFN α-inducible protein 6 (IFI6), IFN α-inducible protein 27-like 2 (IFI27L2), IL-18 receptor 1 (IL18R1), thioredoxin reductase 1 (TXNRD1), and sterile α motif and histidine–aspartate domain-containing protein 1 (SAMHD1).

In total, 17, 286, 18, and 95 mRNAs were uniquely down-regulated and 101, 99, 7, and 56 mRNAs were uniquely up-regulated in the IBV DMV/1639 3 h, IBV DMV/1639 18 h, IBV Mass41 3 h, and IBV Mass41 18 h groups, respectively. Up-regulated mRNAs in the IBV DMV/1639 3 h group included TNFRSF4, TLR21, IRF9, IL-6 (IL6), colony-stimulating factor 3 (CSF3), chemokine ligand (CCL) 4 (CCL4), nucleotide-binding oligomerization domain (NOD)-like receptor family caspase activation and recruitment domain (CARD)-containing (NLRC) 5 (NLRC5), inducible nitric oxide synthase (iNOS or NOS2), and aconitate decarboxylase 1 (ACOD1). FOSB was down-regulated in the IBV DMV/1639 18 h group. Up-regulated mRNAs in the IBV DMV/1639 18 h group included cathepsin S (CTSS) and cluster of differentiation (CD) 38 (CD38). For the IBV Mass41 3 h group, IL-19 (IL19) was down-regulated. Finally, IL-8 (IL8) and transforming growth factor beta receptor III (TGFBR3) were down-regulated and IL-7 (IL7) and C5 were up-regulated in the IBV Mass41 18 h group.

**Figure 5:**
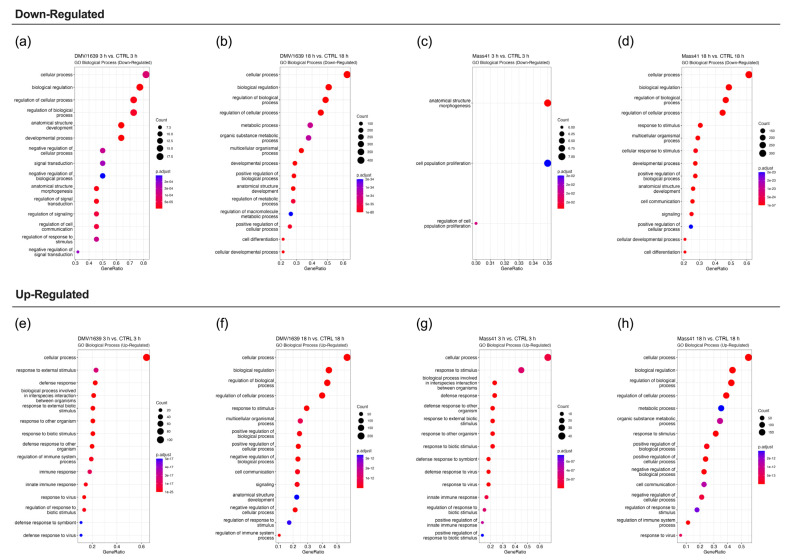
GO functional enrichment analysis for DE mRNAs from cTECs infected with IBV DMV/1639 or IBV Mass41. The dot plots represent the enriched GO Biological Process terms for down-regulated mRNAs from the IBV DMV/1639 at 3 h (**a**), IBV DMV/1639 at 18 h (**b**), IBV Mass41 at 3 h (**c**), and IBV Mass41 at 18 h (**d**) groups, and for up-regulated mRNAs from the IBV DMV/1639 at 3 h (**e**), IBV DMV/1639 at 18 h (**f**), IBV Mass41 at 3 h (**g**), and IBV Mass41 at 18 h (**h**) groups. Count is the number of genes enriched in a GO term and GeneRatio is the percentage of total DE mRNAs in the given GO term. The color intensities represent the adjusted *p*-values. The list of all GO terms for DE mRNAs is found in Table S5.

Figure 5 illustrates the enriched GO terms (BP) for DE RNAs. The full details for the GO enrichment analysis are summarized in Table S5. At the earlier time point, 3 h, GO terms associated with the down-regulated RNAs (Figure 5a,c) tended to be more associated with cell signaling and metabolism, while those associated with the up-regulated RNAs (Figure 5e,g) tended to be associated with defense responses. For example, some of the top GO terms included response to stimulus, regulation of the immune response, and response to virus. At the 18 h time point, the GO terms for down-regulated RNAs (Figure 5b,d) were generally associated with cell signaling and metabolism, or development and cell proliferation. For up-regulated RNAs (Figure 5f,h), GO terms were also associated with defense responses. Pathways are considered enriched when multiple genes from that pathway are up- or down-regulated upon IBV infection.

**Figure 6:**
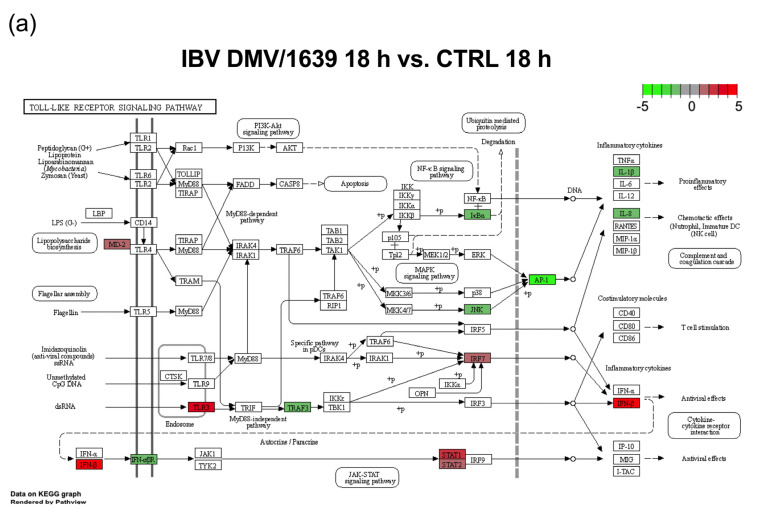

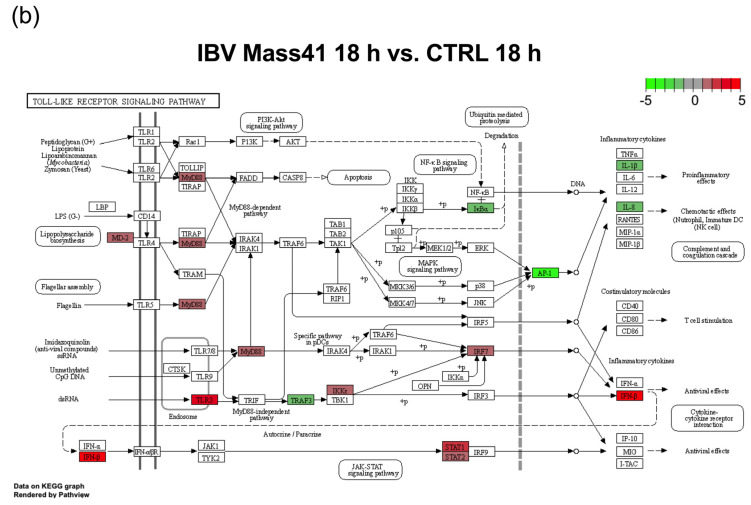
KEGG pathway analysis for DE mRNAs from cTECs infected with IBV DMV/1639 or IBV Mass41. The KEGG pathway figures illustrate the genes within the enriched TLR signaling pathway for IBV DMV/1639 at 18 h (**a**) and for IBV Mass41 at 18 h (**b**). The color intensities represent the log_2_FC. Full details for the pathway analysis are found in Table S5.

All treatment groups, except the IBV Mass41 3 h group, were significantly enriched in immune signaling pathways such as TLR signaling, cytokine–cytokine receptor interaction, RIG-I-like receptor signaling, and cytosolic DNA-sensing. At 3 h, the differences between the enriched pathways of the different strains were marked. The subset of DE genes for the IBV DMV/1639 group was enriched for many pathways, including the ones mentioned above and the NOD-like receptor signaling, calcium signaling, C-type lectin receptor signaling, mitogen-activated protein kinase (MAPK) signaling, and focal adhesion, while the IBV Mass41 group was enriched only for the RIG-I-like receptor signaling pathway. At 18 h, necroptosis was enriched for the IBV DMV/1639 group, while regulation of actin cytoskeleton and TGFβ signaling pathways were enriched for the IBV Mass41 group. The enriched pathways showing the specifically enriched genes for IBV DMV/1639 18 h and IBV Mass41 18 h for the TLR signaling pathway are shown in Figure 6. While many of the DE genes in this pathway are common to both treatment groups, we can observe that, for example, IFN α and β receptor subunit 1 (IFNAR1) and MAPK10, also known as c-Jun N-terminal kinase 3 (JNK3), are down-regulated only in the IBV DMV/1639 18 h group and that inhibitor of nuclear factor κ-B kinase subunit ε (IKBKE) and MYD88 are up-regulated only in the IBV Mass41 18 h group. Full details for KEGG enrichment analysis are summarized in Table S5.

### 3.3. mRNA Expression and Functional Profiles in the Tracheal Tissues of IBV DMV/1639- and IBV Mass41-Infected Chickens

The mRNA expression profiles in tracheal tissues from chickens infected with a high dose (10^5^ EID_50_/bird) of IBV DMV/1639 or IBV Mass41 collected at 4 dpi and 11 dpi were evaluated to determine the effect of the IBV virus strain and collection time point on gene expression. The heatmaps (Figure S2) provided in the Supplementary Files demonstrate the relationships between samples from chickens infected with IBV DMV/1639 at 4 (Figure S2a) and 11 dpi (Figure S2b) or IBV Mass41 at 4 (Figure S2c) and 11 dpi (Figure S2d), relative to their respective control groups (based on differences in mRNA normalized log counts).

For the trachea, the RNA-seq differential expression results are compiled in Table S6. Included in this table are the following comparisons: CTRL 11 dpi vs. CTRL 4 dpi, IBV DMV/1639 4 dpi vs. CTRL 4 dpi, IBV DMV/1639 11 dpi vs. CTRL 11 dpi, IBV DMV/1639 11 dpi vs. IBV DMV/1639 4 dpi, IBV Mass41 4 dpi vs. CTRL 4 dpi, IBV Mass41 11 dpi vs. CTRL 11 dpi, IBV Mass41 11 dpi vs. IBV Mass41 4 dpi. Only the comparisons of the treatment groups and the control groups at the same respective time point were considered (IBV DMV/1639 4 dpi vs. CTRL 4 dpi, IBV DMV/1639 11 dpi vs. CTRL 11 dpi, IBV Mass41 4 dpi vs. CTRL 4 dpi, IBV Mass41 11 dpi vs. CTRL 11 dpi). The results filtered for significantly DE mRNAs (defined by an adjusted *p*-value < 0.05 and a log_2_FC ≥ |1|) are summarized in Table S7.

**Figure 7:**
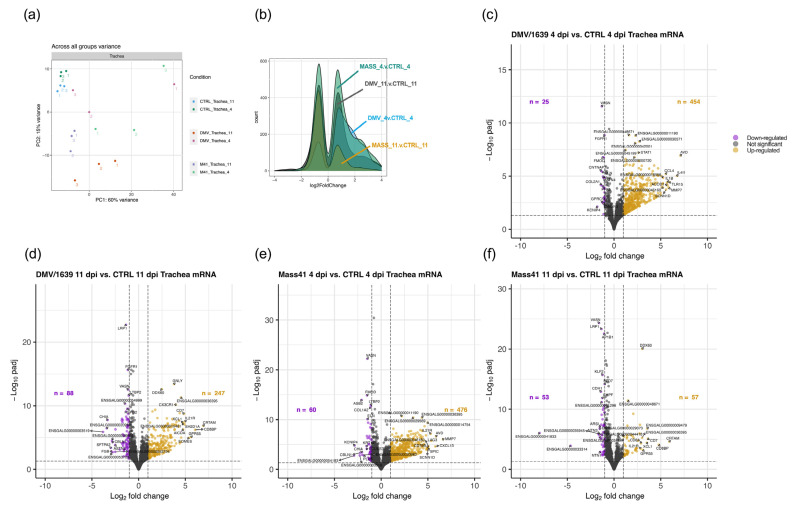
Differential expression of mRNAs in tracheal tissues from chickens infected with IBV DMV/1639 or IBV Mass41. The PCA plot (**a**) evaluates the variance across all samples based on the log counts of all mRNAs. The histogram (**b**) represents the log_2_FC distribution of fluorescence signal intensity ratios for DE mRNAs in tracheal tissues from chickens infected with IBV DMV/1639 or IBV Mass41 at 4 dpi and 11 dpi. The volcano plots show DE mRNAs in tracheal tissues from chickens infected with IBV DMV/1639 at 4 (**c**) and 11 dpi (**d**) or IBV Mass41 at 4 (**e**) and 11 dpi (**f**) relative to their respective control groups. The horizontal dotted line represents the adjusted *p*-value < 0.05 threshold. The vertical dotted lines represent the log_2_FC ≥ |1| (FC ≥ |2|) threshold. The x-axis limits are set from −10 to 10 log_2_FC. Down-regulated mRNAs are represented by purple data points and up-regulated mRNAs are represented by yellow data points. The list of all up- and down-regulated mRNAs for each treatment group are shown in Table S7.

The variance in log counts across all tracheal samples, shown in Figure 7a, demonstrates the differences in normalized log counts between the virus-treated groups and uninfected control groups. Among all treatment groups, including IBV DMV/1639- and IBV Mass41-infected samples at 4 dpi and 11 dpi, a total of 751 DE mRNAs were identified (Table S7). Overall, there are a lower number of down-regulated mRNAs as compared to up-regulated mRNAs at both the 4 dpi and 11 dpi time points for both virus strains (Figure 7b). The numbers of DE mRNAs which passed the adjusted *p*-value < 0.05 and log_2_FC ≥ |1| thresholds were 479 and 335 DE mRNAs, 25 and 88 down-regulated mRNAs, and 454 and 247 up-regulated mRNAs for the IBV DMV/1639 4 dpi and IBV DMV/1639 11 dpi groups, respectively (Figure 7c,d). Furthermore, 536 and 110 DE mRNAs, 60 and 53 down-regulated mRNAs, and 476 and 57 up-regulated mRNAs were identified for the IBV Mass41 4 dpi and 11 dpi groups, respectively (Figure 7e,f).

**Figure 8:**
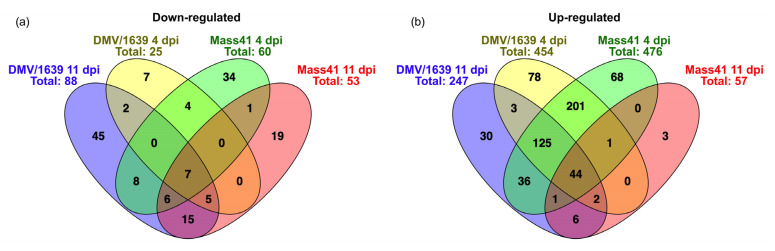
Common and unique DE mRNAs in tracheal tissues from chickens infected with IBV DMV/1639 or IBV Mass41. The Venn diagram shows the common and unique down-regulated (**a**) and up-regulated (**b**) DE mRNAs among tracheal tissues from chickens infected with IBV DMV/1639 or IBV Mass41 at 4 dpi and 11 dpi. Lists of common and unique DE mRNAs are found in Table S8.

Seven down-regulated (Figure 8a) and forty-four up-regulated (Figure 8b) mRNAs were identified in all the treatment groups, for both IBV strains at both time points. Details of the Venn diagram trachea results are summarized in Table S8. The commonly down-regulated mRNAs included contactin-associated protein 1 (CNTNAP1) and fibromodulin (FMOD). On the other hand, the commonly up-regulated mRNAs among all groups included IFI6, MX1, CD8 subunit α (CD8A), CD8 subunit β family member 2 (CD8BP), CD3 δ subunit of T cell receptor complex (CD3D), CD7, IL21R, IL-12 receptor subunit β 2 (IL12RB2), CCL19, CX3C motif chemokine receptor 1 (CX3CR1), C-C chemokine receptor (CCR) 8 (CCR8), chemokine (C motif) ligand (XCL1), STAT1, cytidine/uridine monophosphate kinase 2 (CMPK2), NLRC3, granzyme K (GZMK, ENSGALG00000013546), granzyme A (GZMA), granulysin (GNLY), epithelial stromal interaction 1 (EPSTI1, ENSGALG00000016964), 9L sterile a motif domain-containing 9 like (SAMD9L, ENSGALG00000009479), ζ chain of T cell receptor-associated protein kinase 70 (ZAP70), lymphocyte antigen 6 family member E (LY6E), T cell receptor (TCR) β chain (TCRB, ENSGALG00000014754), cytotoxic and Regulatory T cell molecule (CRTAM), and TCR γ alternate reading frame protein (TARP).

Furthermore, there were 125 mRNAs up-regulated in the IBV DMV/1639 4 dpi and 11 dpi and IBV Mass41 4 dpi groups but not in the IBV Mass41 11 dpi group, which included IRF4, IRF8, STAT4, Burton’s tyrosine kinase (BTK), IFI27L2, Eomesodermin (EOMES), LY96, also known as myeloid differentiation factor 2 (MD-2), IL-2 receptor subunit β (IL2RB), IL-2 receptor subunit γ (IL2RG), IL-4 inducible 1 gene (IL4I1), IL7, IL-7 receptor (IL7R), TNFRSF18, TNFR13B, TNFRSF8, CCL21, CCR2, CCR5, CCR7, C-X-C chemokine receptor (CXCR) 4, CXCR5, C-X-C chemokine ligand (CXCL) 13 (CXCL13), CXCL13-like (CXCL13L) 2 (CXCL13L2), and CD proteins (CD247, CD28, CD38, CD3 ε/CD3E, CD4, CD48, CD72, CD74, CD79 β/CD79B, and CD83). In addition, OASL and DDX60 were the only up-regulated mRNAs shared among the IBV DMV/1639 4 dpi and 11 dpi and IBV Mass41 11 dpi groups, and IFIT5 was the only up-regulated mRNA shared among the IBV DMV/1639 4 dpi and IBV Mass41 4 dpi and 11 dpi groups.

Few similarities in gene expression were observed between the different time points for each IBV strain. For the IBV DMV/1639-infected tissues, there were two commonly down-regulated mRNAs, namely, fibroblast growth factor receptor 1 (FGFR1) and collagen (COL) type XVI α 1 chain (COL16A1, ENSGALG00000026836), and three commonly up-regulated mRNAs, including placenta-associated 8-like 1 (PLAC8L1) and hepatitis A virus cellular receptor 1 (HAVCR1), also known as T cell immunoglobulin. As for the IBV Mass41-infected groups, COL type I α 2 chain (COL1A2) was the only down-regulated mRNA, and no mRNAs were commonly up-regulated at both the 4 dpi and 11 dpi time points.

At 4 dpi, 4 mRNAs were down-regulated, and 201 mRNAs were up-regulated (the largest intersecting group) in both the IBV DMV/1639 and IBV Mass41-infected groups. Up-regulated mRNAs from this group included IRF1, IRF9, TLR1A, TLR2B, TLR3, TLR4, TLR15, IFIH1 (MDA5), IFN-γ (IFNG), IL-1β, IL-22, IL-6, IL-8, IL10RA, IL18R1, IL-18 receptor accessory protein (IL18RAP), IL-1 receptor 2 (IL1R2), IL-20 receptor subunit α (IL20RA), IL-22 receptor subunit α 1 and 2 (IL22RA, IL22RA2), IL8L1, TNFRSF25, TNFRSF4, TNFRSF6B, PARP9, RSAD2 (viperin), MOV10, DTX3L, SAMHD1, NLRC5, TNF α-induced protein 3 (TNFAIP3), TNFAIP3-interacting protein 2 (TNIP2), a disintegrin and metalloproteinase (ADAM) domain 8 (ADAM8), Spi-1 proto-oncogene/hematopoietic transcription factor PU.1 (SPI1), Tyrosine-protein kinase Lyn (LYN), negative regulator of reactive oxygen species (NRROS), ACOD1, CCL4, CD proteins (CD180, CD200R1, CD40 molecule-like family member G/CD40LG, and CD72 antigen/CD72AG), complement components (C1QA, C1QB, C1QC, C1R, and C1S), SOCS1, SOCS3, NFKB inhibitor ε (NFKBIE), and helicase with zinc finger domain 2 (HELZ2). At 11 dpi, there were 15 down-regulated mRNAs, including nuclear receptor subfamily 4 group A member 1 (NR4A1), low-density lipoprotein receptor-related protein 1 (LRP1), and epithelial cadherin (CDH1), and 6 up-regulated mRNAs, including activation-induced cytidine deaminase (AICDA) and synaptotagmin Like 3 (SYTL3), common to the IBV DMV/1639- and IBV Mass41-infected groups.

In total, 7, 45, 34, and 19 mRNAs were uniquely down-regulated and 78, 30, 68, and 3 mRNAs were uniquely up-regulated in the IBV DMV/1639 4 dpi, IBV DMV/1639 11 dpi, IBV Mass41 4 dpi, and IBV Mass41 11 dpi groups, respectively. The 78 up-regulated mRNAs in the IBV DMV/1639 4 dpi group included IRF7, TLR1B, STAT2, CD80, CD300LG, CXCR1, IFI35, TRIM25, TNFSF10, TRAFD1, ZNFX1, MAP3K8, IKBKE, DHX58 (LPG2), and EIF2AK2 (PKR). The 30 up-regulated mRNAs in the IBV DMV/1639 11 dpi group included CXCL13L3 and zinc finger CCCH-type-containing 12D (ZC3H12D). The 68 up-regulated mRNAs in the IBV Mass41 4 dpi group included TLR2A, TLR7, signal-transducing adaptor family member 1 (STAP1), CD1C, CD86, cytotoxic T-lymphocyte associated protein 4 (CTLA4), phospholipase Cg 2 (PLCG2), IL-12 subunit β (IL12B), CCL20, CCR4, and TNFSF11. Finally, the 22 down-regulated mRNAs in the IBV Mass41 11 dpi group included KLF2 and NR4A2.

**Figure 9:**
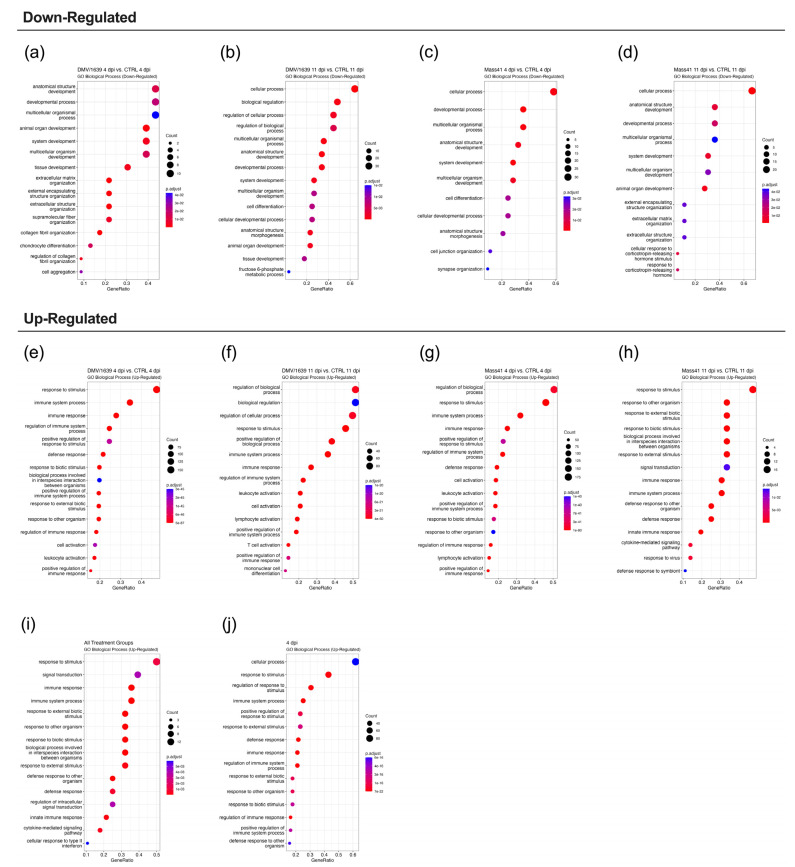
GO functional enrichment analysis for mRNAs in tracheal tissues from chickens infected with IBV DMV/1639 or IBV Mass41. The dot plots represent the enriched GO Biological Process terms for down-regulated mRNAs from the IBV DMV/1639 at 4 dpi (**a**), IBV DMV/1639 at 11 dpi (**b**), IBV Mass41 at 4 dpi (**c**), and IBV Mass41 at 11 dpi (**d**) groups, and for up-regulated mRNAs from the IBV DMV/1639 at 4 dpi (**e**), IBV DMV/1639 at 11 dpi (**f**), IBV Mass41 at 4 dpi (**g**), and IBV Mass41 at 11 dpi (**h**) groups. Enriched GO Biological Process terms for gene subsets common to all treatment groups (**i**) and the 4 dpi groups (**j**) are also shown. Count is the number of genes enriched in a GO term and GeneRatio is the percentage of total DE mRNAs in the given GO term. The color intensities represent the adjusted *p*-values. Full details for mRNA GO enrichment analysis are found in Table S9.

Gene ontology (GO) terms associated with the DE mRNAs revealed functional insights into the gene subsets identified for the different treatment groups (Figure 9). Details of the GO functional analyses for DE mRNAs from tracheal samples are compiled in Table S9. Overall, the down-regulated mRNAs from all infected groups relative to the respective control groups were enriched in BP GO terms mainly related to developmental processes and anatomical structures (Figure 9a–d). On the other hand, the top BP GO terms associated with the up-regulated mRNAs from all groups were related to immune system processes (Figure 9e–j). More specifically, at 4 dpi, the top BP GO terms for both the IBV DMV/1639 (Figure 9e) and IBV Mass41 (Figure 9g) groups included regulation of immune system process, defense response, cell activation, and leucocyte activation. At 11 dpi, in terms of the up-regulated mRNAs from the IBV DMV/1639 group (Figure 9f), the top BP GO terms included lymphocyte activation, leucocyte activation, and T cell response. For the up-regulated mRNAs from the IBV Mass41 (Figure 9h) group, the top BP GO terms included defense response, innate immune response, and cytokine-mediated signaling. Furthermore, the top enriched BP GO terms for up-regulated mRNAs found in all treatment groups (Figure 9i) in the 4 dpi groups only (Figure 9j) were associated with immune system processes and defense response.

**Figure 10:**
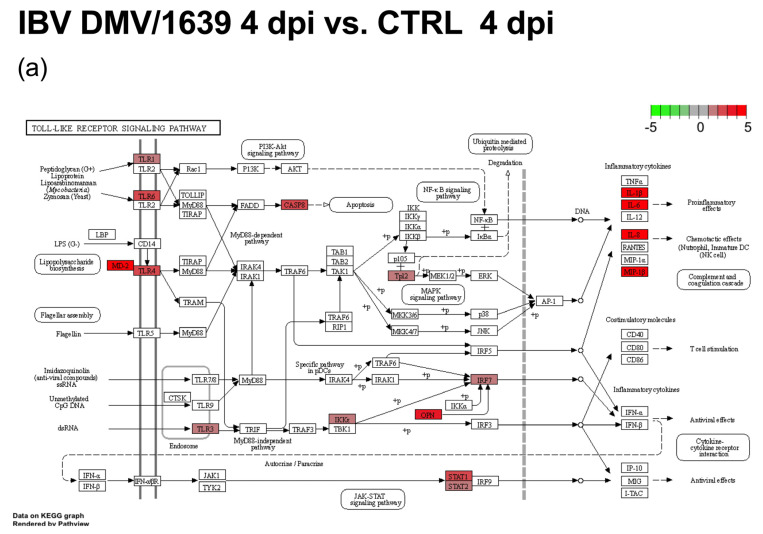

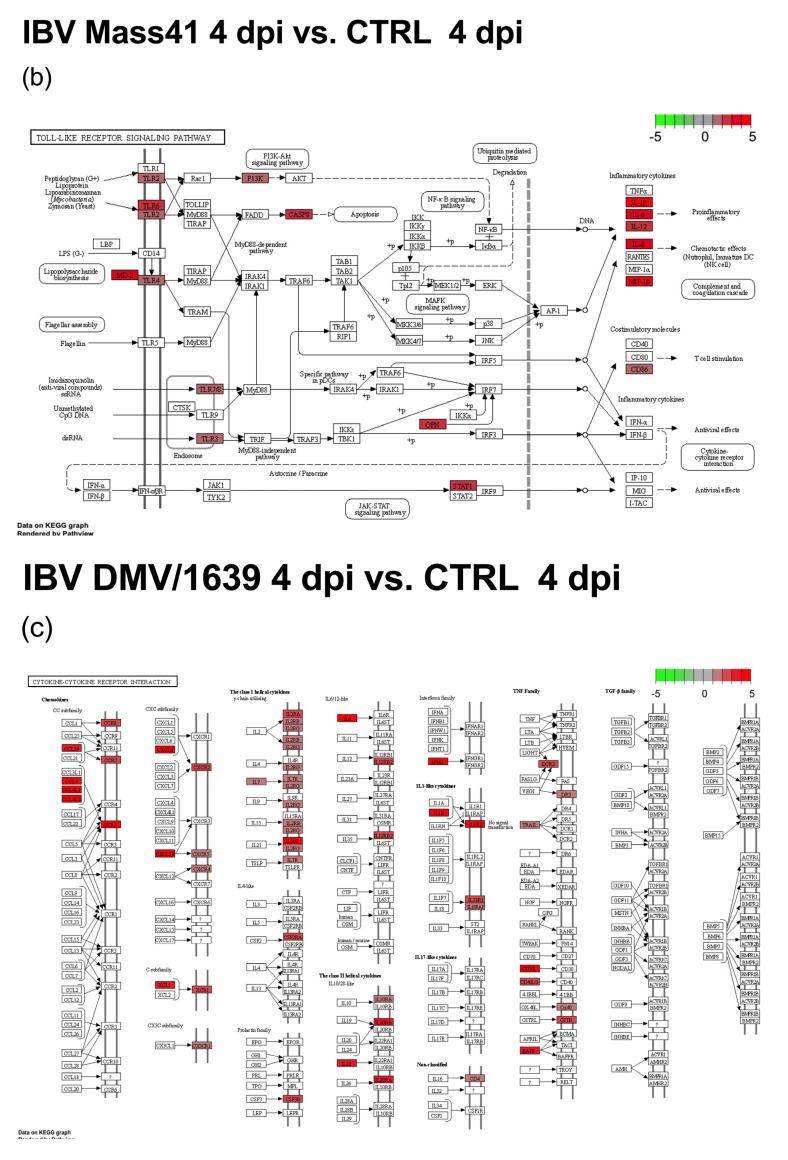

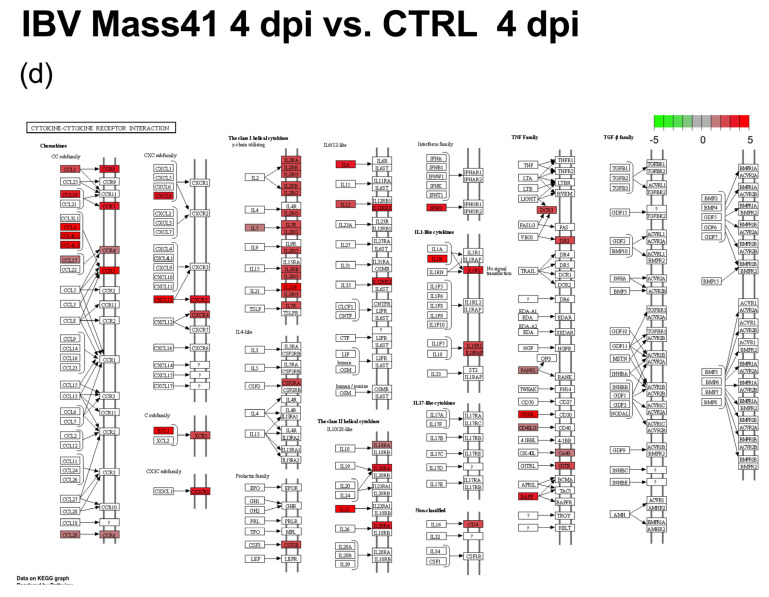
KEGG pathway analysis for DE mRNAs in tracheal tissues from chickens infected with IBV DMV/1639 or IBV Mass41. DE genes in the enriched KEGG pathways are shown for IBV DMV/1639 4 dpi TLR signaling (**a**), IBV Mass41 4 dpi TLR signaling (**b**), IBV DMV/1639 4 dpi cytokine-cytokine receptor interaction (**c**), and IBV Mass41 4 dpi cytokine-cytokine receptor interaction (**d**). KEGG pathway analysis figures were generated using the R package pathview. The color intensities represent the expression levels of the DE mRNAs identified in the RNA-seq analysis. Full details for mRNA KEGG enrichment analysis are found in Table S9.

Upon further KEGG pathway analysis, all treatment groups were found to be enriched for the cytokine–cytokine receptor interaction and cell adhesion molecule pathways (Table S9). The enriched pathways for both 4 dpi groups included TLR signaling, necroptosis, NOD-like receptor signaling, retinoic acid-inducible gene I (RIG-I)-like receptor signaling, apoptosis, and cytosolic DNA sensing. The p53 signaling pathway was enriched for the IBV DMV/1639 4 dpi group only, while the regulation of actin cytoskeleton and focal adhesion pathways was enriched for the IBV Mass41 4 dpi group only. The IBV DMV/1639 11 dpi group was also enriched for cell adhesion molecules, endocytosis, and C-type lectin receptor signaling pathways, while the IBV Mass41 11 dpi group for cell adhesion molecules and extracellular matrix (ECM)–receptor interaction pathways. Enrichment and expression of the specific components in the TLR signaling (Figure 10a,b) and cytokine–cytokine receptor interaction (Figure 10c,d) pathways are shown for the IBV DMV/1639 4 dpi and IBV Mass41 4 dpi treatment groups. The pathway enrichment analysis revealed that several DE genes are common to both 4 dpi groups, but some important differences are observed. For example, IKBKE, IRF7, and STAT2 are up-regulated in the IBV DMV/1639 4 dpi group but not in the IBV Mass41 4 dpi group.

### 3.4. Comparisons of DE mRNAs in In Vitro and In Vivo Infection Models

Overall, upon comparing the expression patterns in in vitro and in vivo RNA-seq datasets, a total of 162 DE mRNAs were found to be common to both infection models in at least one treatment group (Table S10). In total, 21 of these DE mRNAs were down-regulated and 141 were up-regulated. The down-regulated mRNAs included kelch-like family member 30 (KLHL30), KLF2, NR4A1, and NR4A2. Up-regulated mRNAs included SAMHD1, NLRC5, TRAFD1, IL18R1, IL-6, IRF7, IRF1, ACOD1, TRIM25, CCL4, DDX60, DHX58 (LPG2), TLR3, STAT1, STAT2, PARP9, IFIH1 (MDA5), CD38, LY96 (MD-2), SOCS1, RSAD2 (viperin), EIF2AK2 (PKR), OASL, MX1, CMPK2, IFIT5, and sodium channel epithelial 1 subunit δ (SCNN1D).

**Figure 11:**
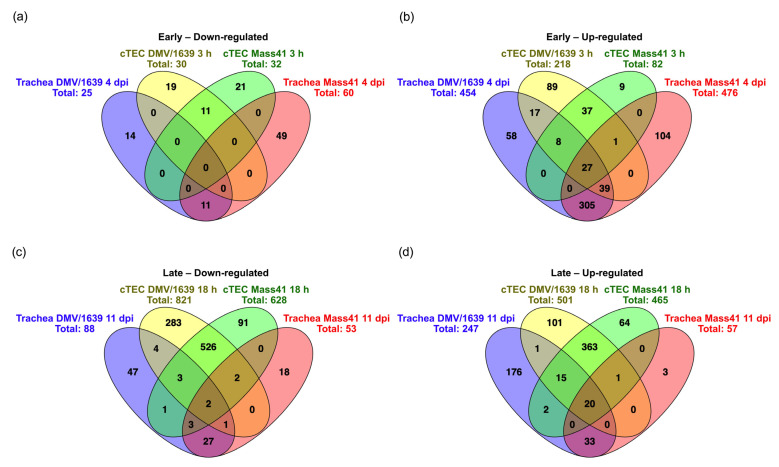
Common and unique DE mRNAs from cTECs and the trachea in the context of IBV DMV/1639 or IBV Mass41 infection at early and late time points post-infection. The Venn diagram illustrates common and unique down-regulated (**a**) and up-regulated (**b**) mRNAs at the early time points post-infection and down-regulated (**c**) and up-regulated (**d**) at the late time points post-infection. Lists of common and unique DE mRNAs are found in Table S10.

More specifically, Figure 11 illustrates the gene overlaps at the early time points post-infection, 3 h (in vitro), or 4 dpi (in vivo), for down-regulated (Figure 11a) and up-regulated (Figure 11b) mRNAs and at the late time points post-infection, 18 h or 11 dpi, for down-regulated (Figure 11c) and up-regulated (Figure 11d) mRNAs, for both IBV infection models. At the earlier time points post-infection, we did not observe any overlap with down-regulated mRNAs; however, 27 up-regulated mRNAs were common to all early treatment groups, including TLR3, IFIT5, IFIH1 (MDA5), MX1, RSAD2 (viperin), CMPK2, SOCS1, and SCNN1D. Two mRNAs were down-regulated in all treatment groups at the later time points post-infection, namely, CNTNAP1 and NR4A1, while twenty mRNAs were up-regulated in all later time point treatment groups, including IFI6, LY6E, MX1, OASL, STAT1, and CMPK2.

Although we observed these important overlaps in gene expression among the two infection models, 938 down-regulated mRNAs and 567 up-regulated mRNAs were identified in the IBV-infected cTECs only. Down-regulated mRNAs included SLC6A4, PPP4R4, TRAF3, JUN, and NFKBIA. Up-regulated mRNAs included IFITM5, ADAR, and MOV10. In contrast, we observed 132 down-regulated mRNAs and 457 up-regulated mRNAs in the IBV-infected tracheal tissues only. COL1A2, COL2A1, COL16A1, elastin (ELN), and LRP1 were among the down-regulated mRNAs and TLR4, TLR7, CCR2, CCL17, CCL20, IL-22, NLRC3, IFN-γ, GZMA, and GNLY were among the up-regulated mRNAs. Finally, we observed some cases of differential dysregulation for certain mRNAs in vitro versus in vivo. For example, CDH1 was up-regulated in the cTEC IBV DMV/1639 and IBV Mass41 18 h groups, but down-regulated in the trachea IBV DMV/1639 and IBV Mass41 11 dpi groups. IL-1β and SOCS3, on the other hand, were down-regulated in the cTEC IBV DMV/1639 and IBV Mass41 18 h groups and up-regulated in the tracheas of IBV DMV/1639- and IBV Mass41-infected 4 dpi groups.

### 3.5. Host mRNA Gene Expression Validation

To validate the RNA-seq results, qPCR was performed to detect expression of a subset of DE mRNAs using tracheal samples from IBV DMV/1639-infected chickens. Three down-regulated and four up-regulated mRNAs were selected, and the relative expression of these genes was measured (Table 1). The qPCR results demonstrate that the patterns of host mRNA expression are similar to the patterns determined by RNA-seq, with little variation in the magnitude of the expressions.

## 4. Discussion

Understanding the different factors which can affect the underlying mechanisms of IBV pathogenesis, particularly at the primary site of infection, airway epithelial cells, is key in developing new strategies for IBV control. In the present study, we aimed to characterize the induction of the antiviral response following IBV infection in vitro and in vivo. We expected that IBV infection would impact the overall induction and initiation of the host immune responses and wanted to investigate the specific factors and mediators involved. First, we demonstrated that IBV DMV/1639 and IBV Mass41 replicate in cTECs in vitro and in the trachea in vivo and induce strain- and time-dependent expression of host mRNAs. Second, these observations also provided insight into the regulation of expressed transcripts involved in immune system signaling pathways upon IBV infection of cTECs and the trachea. Finally, we demonstrated the differences in gene expression patterns between in vitro and in vivo tracheal IBV infection models.

Tracheal organ culture has long been used to investigate IBV infection [106,107,108,109,110,111,112]. While this ex vivo model offers many benefits, understanding the mechanisms specifically at the level of tracheal epithelial cells is useful for studying immediate host responses under highly controlled conditions. Our findings shed light on the replication dynamics of IBV in cTECs, providing valuable insights into host–pathogen interactions under specific conditions. Both IBV DMV/1639 and IBV Mass41 strains demonstrated a comparable replication capability in this in vitro model. Few studies have evaluated IBV infection in cTEC models, despite the significance of airway epithelial cells as the primary target for IBV during initial infection. Shen and colleagues established a primary cTEC culture system as a means to study viral cytopathogenesis and showed that these cells were susceptible to IBV Taiwan (TW)-type infection [65]. In addition, Kint and colleagues infected cTECs with IBV Mass41-type to demonstrate the delayed induction of the type I IFN response [36]. The latter two studies did not provide data of IBV replication kinetics that can be used for comparison of our IBV replication kinetic data. Although the cTECs were closely monitored for growth and viability over the course of the experiments, a cell viability assay to confirm our visual observations would be an important addition for future work.

Generally, the in vivo inoculation doses of IBV used in this study, low (10^4^ EID_50_/bird) and high (10^5^ EID_50_/bird), did not have an impact on the viral genome load detected in the trachea. This 10-fold difference in IBV inoculation dose may not be enough to see a difference in resulting viral genome load in the trachea. Several similar studies infecting young or adult chickens with IBV use an inoculation dose of 10^6^ EID_50_/bird [24,113,114] and we may have seen greater differences between the groups if this upper limit had been used for the high dose. Typically, the highest concentration of IBV is found in the trachea at 3–5 dpi; however, IBV has been detected as early as 3 dpi in various tissues [115]. As a result, we chose the 4 dpi time point and a later time point of 11 dpi for sample collection. Given that the upper respiratory tract of the chicken is known to mount strong innate antiviral responses against invading respiratory pathogens [6,64,116], the significant decrease (*p*-value < 0.05) in viral genome load from 4 dpi to 11 dpi in the trachea for the high-dose IBV DMV/1639 and low-dose IBV Mass41 groups may indicate the dissemination of the virus to establish infection and persist at distal sites.

Previously, RNA-seq analyses have been conducted studying the interaction between IBV strains such as Beaudette, Mass41 strains [49], and K047-12 [51] in chicken kidney cells and focused on only one time point following IBV inoculation. Our data are different since we focused on cTECs involved at the IBV entry site (respiratory mucosa) and included an additional IBV strain which has recently become endemic in North America, IBV DMV/1639 [21,22]. Furthermore, we included an early time point and a later time point for both of our in vitro and in vivo studies, which allowed us to observe changes in host transcripts over the course of IBV infection in different models. The mRNA expression profiles of IBV DMV/1639- or IBV Mass41-infected cTECs or tracheas provide evidence that there are distinct interactions between the IBV strains and the host. Collection time points further separate expression profiles, indicating a switch in gene expression from a naïve to activated antiviral state.

Dinan and colleagues observed 579 up-regulated and 132 down-regulated genes in response to IBV Beaudette and Mass41 strains in kidney cells 24 h following infection [49], whereas Lee and colleagues observed 787 up-regulated and 297 down-regulated genes in response to IBV K047-12 infection in kidney cells 48 h following infection [51]. In comparison, we observed 30 (3 h) and 821 (18 h) down-regulated genes, and 218 (3 h) and 501 (18 h) up-regulated genes, for IBV DMV/1639 infection of cTECs and 32 (3 h) and 628 (18 h) down-regulated genes, and 82 (3 h) and 465 (18 h) up-regulated genes, for IBV Mass41 infection of cTECs. Another 2013 microarray study by Cong and colleagues determined that IL6, STAT1, MYD88, and IRF1, all of which were present in our IBV-infected cTEC data, were key genes in chicken kidneys during IBV infection [47]. Our data show more genes are turned on or off as the infection progresses from 3 h to 18 h in cTECs following IBV DMV/1639 or IBV Mass41 infection. This is expected, as viral infection disturbs the host homeostasis, triggering the activation of several downstream signaling pathways and factors involved in host defense against the invading pathogen [117,118].

Downstream of TLR and ligand engagement, two pathways can be activated: MYD88-dependent and MYD88-independent pathways [119]. In the current study, we observed that the MYD88 gene is enriched following IBV DMV/1639 infection at 3 h and IBV Mass41 infection at 18 h. Previously, it has been shown that IBV infection in kidneys and trachea up-regulates MYD88 expression [47,68]. Up-regulation of IRF7, which is expressed downstream of both MYD88-dependent and MYD88-independent pathways, was evident following IBV infection in cTECs. This agrees with the previous observation in tracheas of resistant and susceptible lines of chickens following IBV infection [43]. One of the antiviral cytokines enriched during IBV infection in cTECs is IFNβ and this cytokine is up-regulated downstream of TLR3 and IRF7 activation [120,121]. Our data provide evidence that IBV infection also up-regulates TLR3 and IRF7 genes in cTEC.

KEGG pathway analyses at the later time point (18 h) indicating enrichment for the innate immune response, particularly for the TLR signaling pathway following infection with IBV DMV/1639 or IBV Mass41, is not surprising. The replication of IBV in cTECs leads to availability of double-stranded RNA intermediates within cTECs (TLR3 ligand) and the increased TLR3 we observed has been recorded in trachea following IBV infections [38,68]. The increased gene expression of TLR21 following IBV DMV/1639 infection of cTECs at 3 h is difficult to explain since the TLR21 ligand is CpG (cytosine followed by guanine residues) DNA and IBV is an RNA virus [122,123]; however, there is evidence that CpG DNA can activate the innate immune response to suppress IBV replication in ovo [124], which suggests this sensor may play an unknown role during viral infection. In Lee and colleague’s IBV work in kidney cells, up-regulation of TLR7 has been recorded at 48 h following infection [51]. However, we did not see TLR7 up-regulation with IBV strains in cTECs and, potentially, this discrepancy may be related to the IBV strain used and differences in host cells and observed time points.

On the other hand, in the trachea, we observed 25 (4 dpi) and 88 (11 dpi) down-regulated mRNAs, and 454 (4 dpi) and 247 (11 dpi) up-regulated mRNAs, for the IBV DMV/1639 group, and 60 (4 dpi) and 53 (11 dpi) down-regulated mRNAs, and 476 (4 dpi) and 57 (11 dpi) up-regulated mRNAs, for the IBV Mass41 group in vivo. Smith and colleagues identified several important DE genes in IBV Mass41-infected tracheal tissues from susceptible and resistant birds, such as TLR3, IRF7, STAT1, IFIH1 (MDA5), MX1, IFIT5, and OASL, which were also up-regulated in our IBV-infected tracheal tissue data [43]. Ghobadian and colleagues indicated that the Iranian variant-2-like IBV strain IS/1494 induced variable host gene expression in different chicken hybrid tracheal tissues but also demonstrated the importance of certain genes such as TLR3, IFIH1 (MDA5), and IRF7 and the enrichment of the TLR signaling pathways [45]. Many of the important genes mentioned in the studies above and found in our DE gene data emphasize the importance of ISGs. For example, in chickens, IFIT5 is expressed downstream of IFNβ expression following IBV infection in kidneys [125] and is known to sequester viral RNA impacting viral replication [126]. In other host–viral models, it has been observed that IFIT5 induces innate responses effective against viral infections [127].

Similar to the results for our cTEC data, KEGG pathway analyses indicated enrichment for the innate immune response. Once again, the enrichment of the cytokine–cytokine interaction pathway for all in vivo treatment groups is not surprising given that the significant involvement of pro-inflammatory cytokines during IBV infection has been well documented [7,128,129,130,131].

There is a large interest in understanding IBV immunopathogenesis in reproductive organs due to the detrimental impact of certain strains, including IBV DMV/1639 and IBV Mass-type strains, on the reproductive tracts. The strains used in our study are different in terms of their pathogenesis and specific tissue tropism. Decoding the mechanisms during initial infection may help to explain these differences. Recently, Farooq and colleagues showed that tracheal lesions in IBV Mass-type-infected chickens are more severe than those in IBV DMV/1639-infected chickens, while misshaped eggs or eggs with soft shells were only observed with IBV DMV/1639-infected chickens [59]. The differences in gene expression observed for our different strains may be related to the variable aspects of pathogenicity observed. For example, for cTECs at 3 h, the IBV DMV/1639 group is enriched in a higher number of immune signaling pathways compared to the IBV Mass41 group. Moreover, TGFβ signaling has several roles, including in re-epithelization and inflammation [132] and the enrichment of TGFβ signaling in the IBV Mass41 18 h group, but not the IBV DMV/1639 18 h group, may explain the difference in severity of the tracheal lesions mentioned above. Similarly, for the tracheal tissues at 11 dpi, the IBV DMV/1639 group is associated with more immune signaling pathways compared to the IBV Mass41 group, supporting what was observed in cTECs in vitro. It is important to acknowledge that the differences in enriched KEGG pathways is somewhat dependent on the different number of DE genes between the treatment groups, which may introduce a potential bias, and that the variable number of DE genes may be due small differences in replication kinetics in host cells or tissues. Furthermore, this is the first transcriptomics study evaluating the mRNA expression profiles during IBV DMV/1639 infection and future studies are needed to evaluate the expression profiles in different tissues and at other time points of infection.

While our in vitro and in vivo models for IBV infection provided insights into mRNA host response regulation in their own respect, this study also allows us to have a head-to-head comparison of the infection models for the same strains. Overall, there were 1653 DE mRNA in cTECs and 751 DE mRNA in the trachea for all treatment groups. Although cell culture systems are considered reliable platforms for studying anything from cell behavior to detailed molecular mechanisms, it is not surprising that we see differences between our infection models at different time points. The most evident difference between our models is that the tracheal tissues are a mix of different cells and connective tissues, while the cTEC model is a monolayer of isolated tracheal epithelial cells. It has long been known that the modulation of gene expression in vitro versus in vivo is distinct [133]. In the in vivo model, we reported the down-regulation of ECM components such as collagens and elastin. As the main fibers of the ECM, these components are important for the structural support in cells and tissues [134] and are linked to the regulation of epithelial cell function [135]. The down-regulation of these ECM genes in the trachea may be explained by the IBV-induced epithelial changes in the respiratory tract resulting in a loss of ciliary activity and tracheitis [2]. Furthermore, we observed up-regulated IFN-γ, a type II IFN, in the trachea in vivo. IFN-γ leads to the activation of the antiviral response through the Janus kinase (JAK–STAT signaling pathway [136]. Kameka and colleagues showed an initial IBV-induced down-regulation of IFN-γ in the trachea and lungs of chickens [68] and Ma and colleagues reported that IBV nsp14 targets JAK1 to inhibit JAK-STAT signaling in chicken macrophages, but also highlighted the importance of IFN-γ anti-IBV activity through the induction of ISG expression [137], suggesting that the increase in IFN-γ expression in the trachea may play a role in the antiviral response in the upper respiratory tract.

Furthermore, infection and sample collection time points are vastly different based on the nature of the model systems. These expression profiles can only give us a snapshot in time as the antiviral response against IBV progresses. Nevertheless, 21 down-regulated mRNAs and 141 up-regulated mRNAs are common to both the cTEC and trachea infection models. TLR3, IFIH1 (MDA5), SOCS1, OASL, DDX60, STAT1, MX1, CMPK2, LY96 (MD-2), STAT1, STAT2, TRIM25, IRF7, and IFIT5 are among the up-regulated genes, many of which have been identified as key genes in previous transcriptomic IBV studies mentioned above. In addition, SCNN1D was commonly up-regulated for all treatment groups across the cTEC and trachea data. In multi-ciliated cells, the epithelial sodium channel is located in cilia and plays an essential role in the regulation of epithelial surface liquid volume necessary for cilial transport of mucus [138]. In our study, the up-regulation of SCNN1D potentially contributes to enhanced mucous production in the trachea and the upper respiratory tract following IBV infection [114,139,140,141]. Finally, MX1 and CMPK2 are up-regulated in all treatment groups across both in vitro and in vivo studies. MX1 is an ISG known to have antiviral activity against a wide range of RNA viruses [142,143]. CMPK2, on the other hand, can act as a host restriction factor to inhibit the replication of coronaviruses, including IBV [144,145].

RNA-seq is a powerful tool and host transcriptomic data can be used to evaluate the effect of pathogen variants on the host mRNA signature to identify key hallmarks of the resulting disease [146]. With IBV, characterizing the expression of specific host antiviral factors may be useful for monitoring the disease and distinct pathogenesis induced by different IBV strains. Additional studies are needed, but the differences in gene expression induced by IBV DMV/1639 and IBV Mass41 in this study could be correlated with the well-documented differences in pathogenesis [24,59,147]. Taken together, these host mRNA expression profiles provide an overview of the response to IBV infection. Furthermore, we identified key genes that may play a role in regulating IBV infection. In future studies, these candidate genes must be verified at the protein expression level by conducting proteomics screening studies, for example. Furthermore, the specific functions of these candidate genes could be assessed by silencing their expression through RNA interference (RNAi) experiments in the context of IBV infection, followed by validation of these results in vivo. This work would help to correlate differential gene expression with strain-specific tissue tropism, virulence, and immune responses observed both in the laboratory and field settings. Overall, this study provides a useful framework for examining IBV infection in tracheal epithelial cells, which could have significant implications for understanding and treating viral respiratory infections.

## 5. Conclusions

Transcriptomic data revealed important patterns of expression key to uncovering relevant factors in host responses during infection. We reported a total of 248, 1322, 114, and 1093 DE mRNAs for IBV DMV/1639 at 3 h, IBV DMV/1639 at 18 h, IBV Mass41 at 3 h, and IBV Mass41 at 18 h post-infection, respectively, and a total of 479, 335, 536, and 110 DE mRNAs for the IBV DMV/1639 4 dpi, IBV DMV/1639 11 dpi, IBV Mass41 4 dpi, and IBV Mass41 11 dpi groups, respectively. The findings provide insights into strain-specific and temporal-related changes in gene expression, which could be valuable in understanding the molecular mechanisms underlying IBV infection.

We identified important genes DE in both our in vitro and in vivo infection models consistent with previous studies, namely, TLR3, IFIH1 (MDA5), SOCS1, OASL, DDX60, STAT1, MX1, CMPK2, LY96 (MD-2), STAT1, STAT2, TRIM25, IRF7, and IFIT5. Furthermore, we characterized key variations in gene expression in the trachea unique to the in vivo model, such as changes in collagen, elastin, TLR4, TLR7, CCR2, CCL17, and IFN-γ expression. Future studies should confirm expression of these genes at the protein level. Overall, the study highlights the complexity of host–virus interactions and emphasizes the importance of investigating gene expression changes over time to uncover the dynamics of the infection process.

## Figures and Tables

**Table 1:** Comparison of host mRNA expression fold-changes (FC) of selected genes between RNA-seq and qPCR in tracheas from IBV DMV/1639-infected chickens relative to the uninfected control group at 4 dpi and 11 dpi.

		IBV DMV/1639 4 dpi		IBV DMV/1639 11 dpi	
	Gene	RNA-Seq FC	qPCR FC	RNA-Seq FC	qPCR FC
Down-regulated	KLHL30	−1.012	−3.230	−1.147	−1.826
	FMOD	−2.283	−2.987	−2.181	−1.401
	NR4A1	−1.005	−3.131	−2.516	−2.829
Up-regulated	SOCS1	6.538	3.030	1.057	1.633
	TLR3	2.192	1.697	1.704	1.061
	STAT1	6.167	1.636	2.635	1.168
	STAT2	2.749	4.224	1.141	2.269

## Data Availability

All relevant data are provided within the paper and the Supplementary Files or available upon request. The datasets generated during this study are available in the Sequence Read Archive (SRA), BioProject PRJNA1088470.

## Supplementary Materials

The following supporting information can be downloaded at: https://www.mdpi.com/article/10.3390/v16040605/s1, Figure S1: cTEC_heatmaps; Figure S2: trachea_heatmaps; Table S1: Primers_for_qPCR; Table S2: All_DEGs_mRNA_cTEC_in_vitro; Table S3: Filtered_DEGs_mRNA_cTEC_in_vitro; Table S4: venn_results_cTEC_in_vitro_mRNA; Table S5: GO_KEGG_mRNA_cTEC_in_vitro; Table S6: All_DEGs_mRNA_trachea_in_vivo; Table S7: Filtered_DEGs_mRNA_trachea_in_vivo; Table S8: venn_results_trachea_in_vivo_mRNA; Table S9: GO_KEGG_mRNA_trachea_in_vivo; Table S10: cTEC_trachea_all_mRNA_overlap.
