# Supplementary figures and images for: Host Immune Response Modulation in Avian Coronavirus Infection: Tracheal Transcriptome Profiling In Vitro and In Vivo
Source: Viruses. 2024 Apr 14;16(4):605. doi: 10.3390/v16040605 (PMC11053446; doi:10.3390/v16040605)

(a)

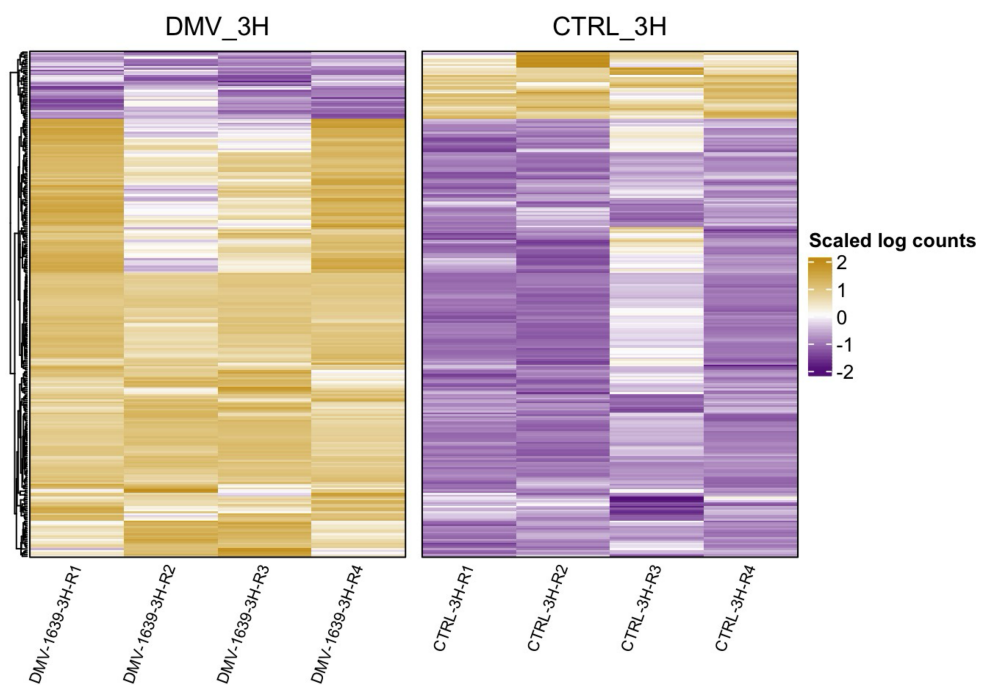

(b)

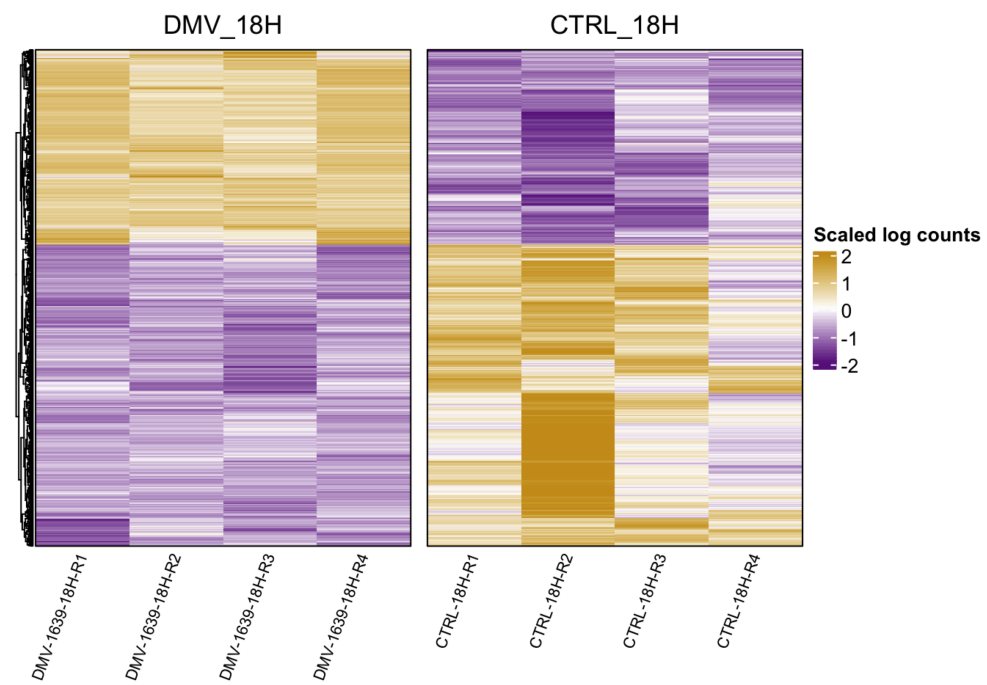

(c)

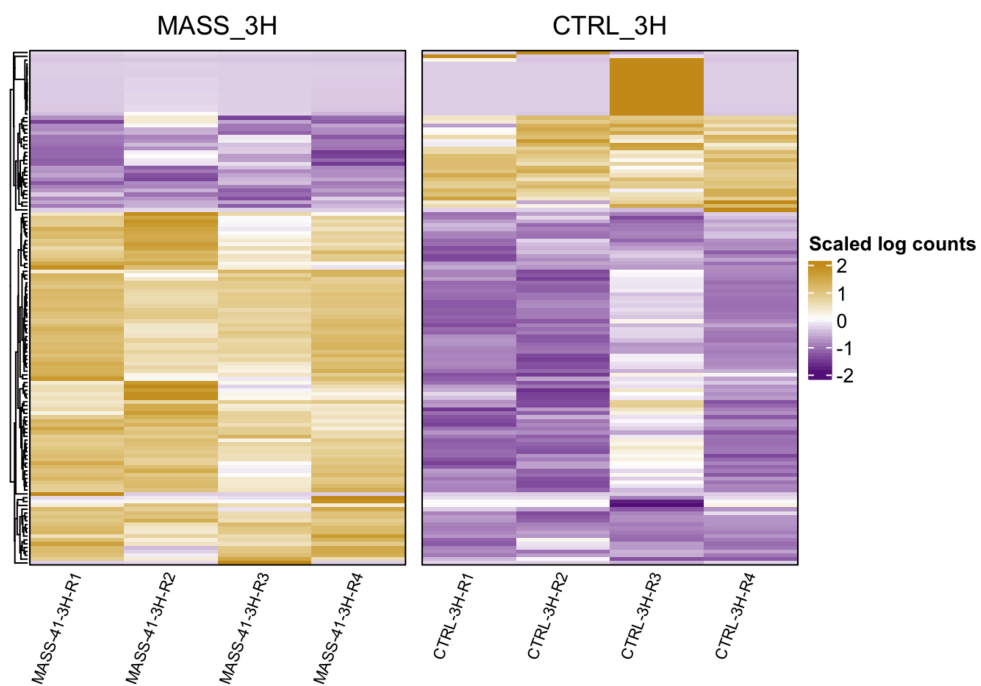

(d)

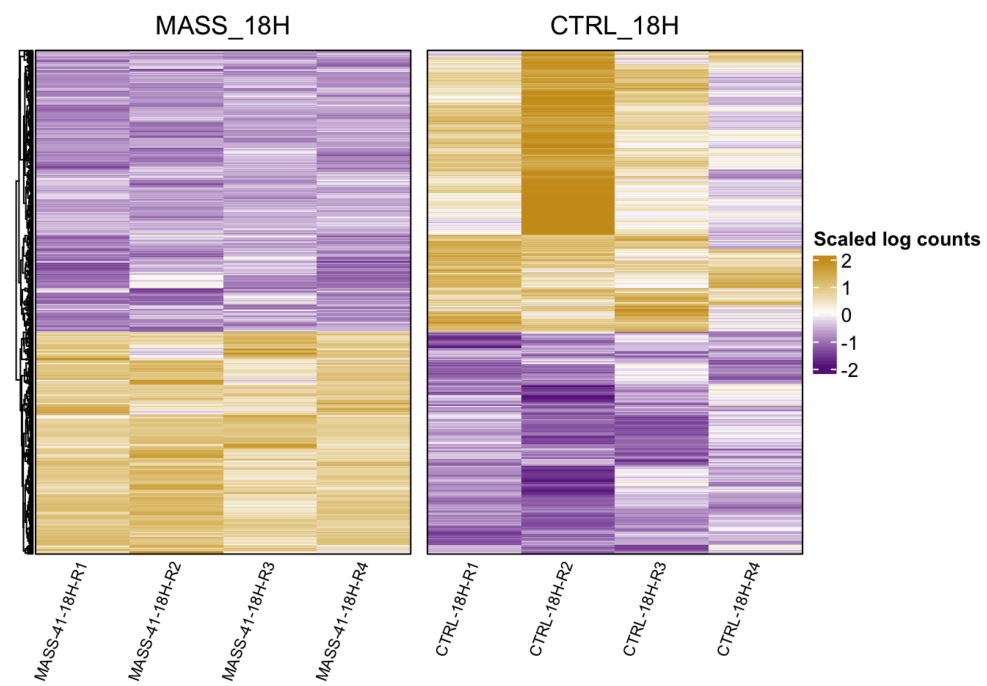

Supplement: Supplementary file 1 [file viruses-16-00605-s001.zip › Fig_S1_cTEC_heatmaps.pdf]

(a)

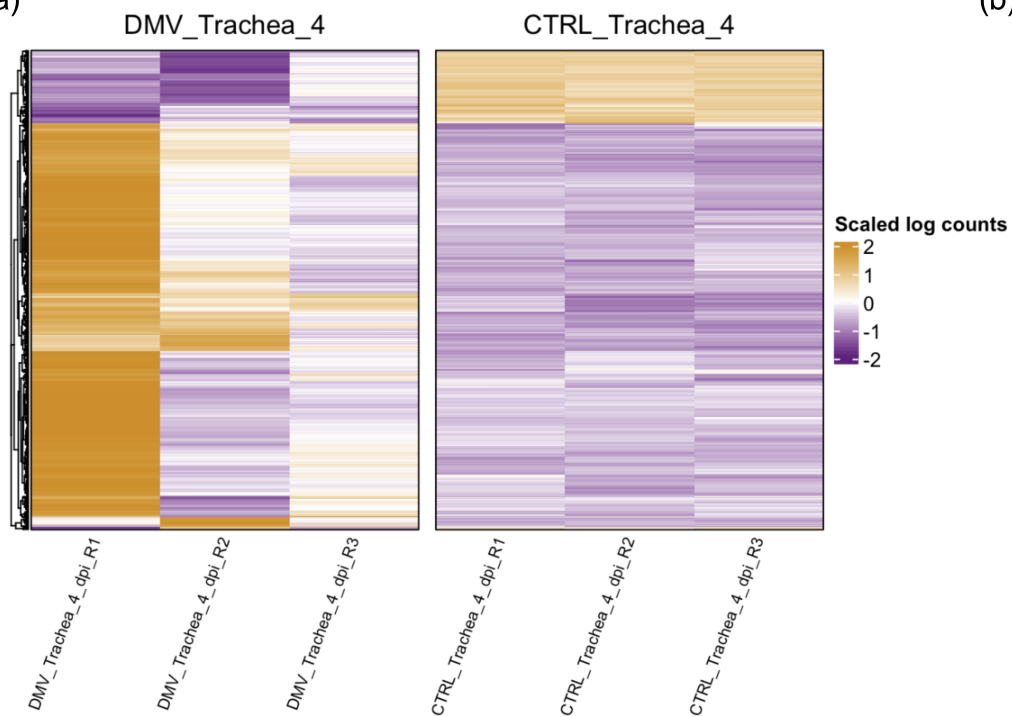

(b)

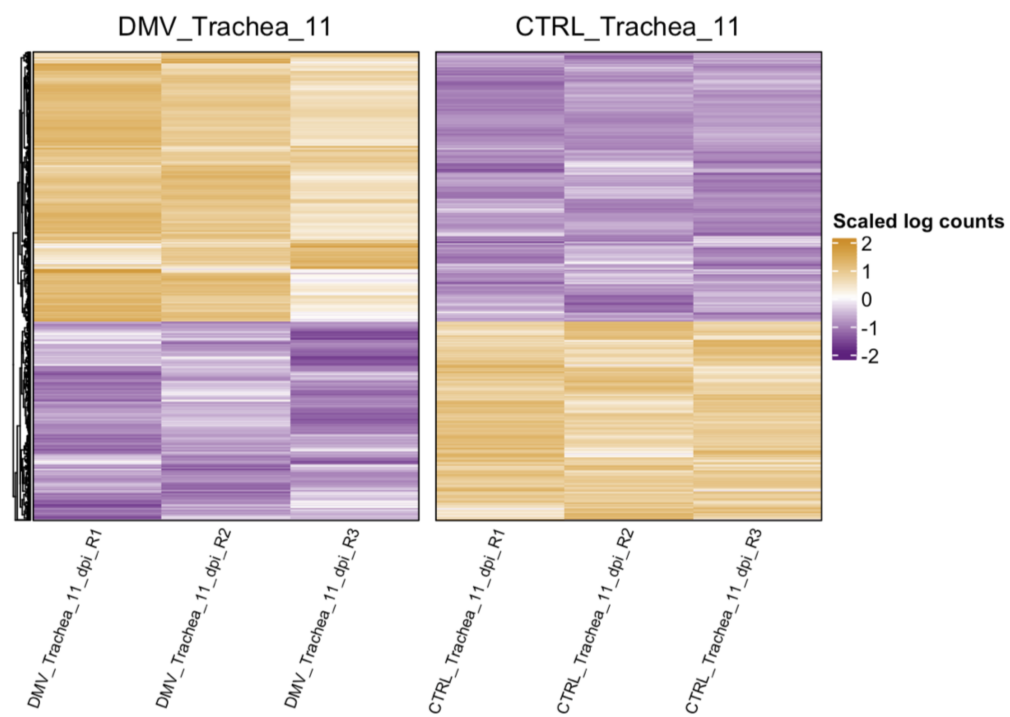

(c)

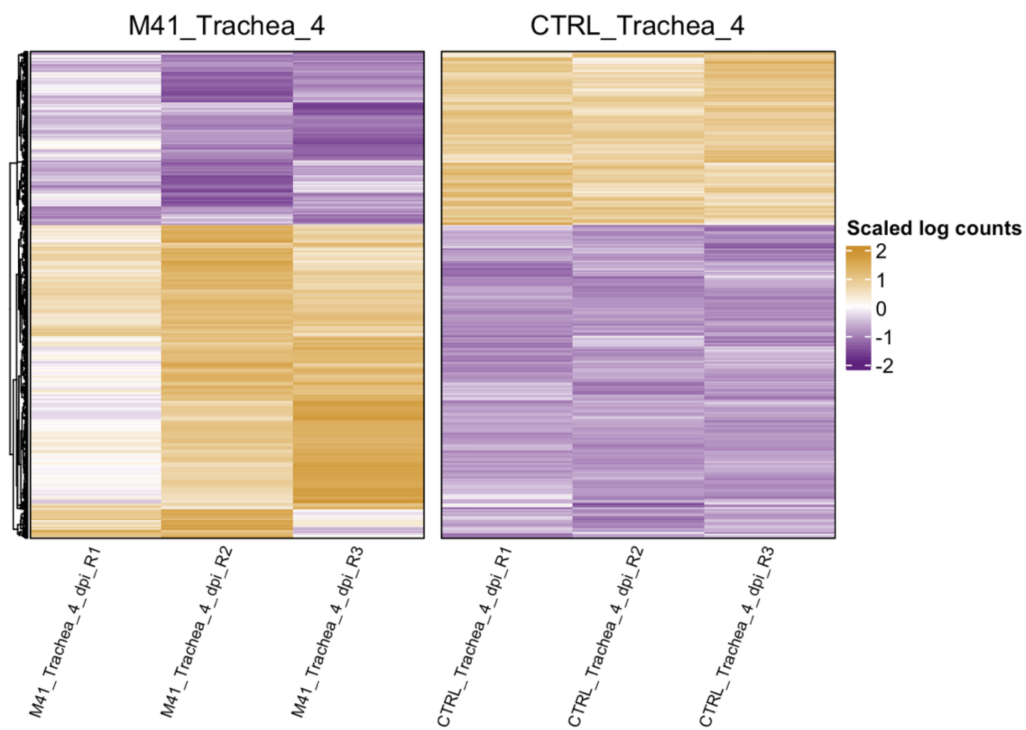

(d)

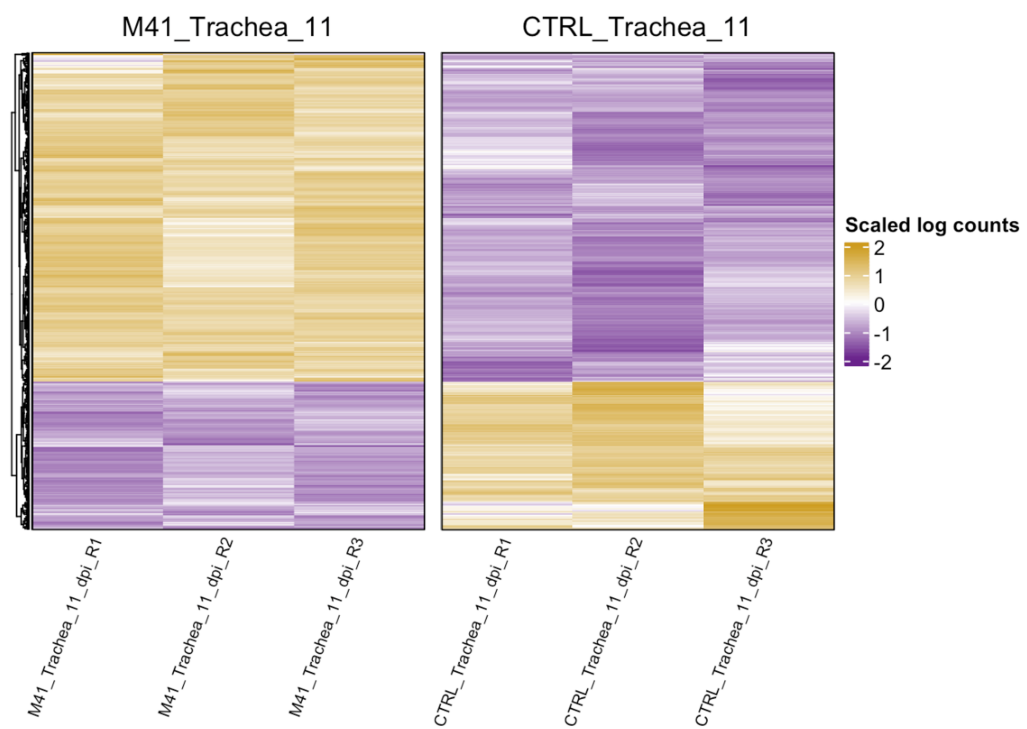

Supplement: Supplementary file 1 [file viruses-16-00605-s001.zip › Fig_S2_trachea_heatmaps.pdf]
